# Is level of implementation linked with intervention outcomes? Process evaluation of the *TransformUs* intervention to increase children’s physical activity and reduce sedentary behaviour

**DOI:** 10.1186/s12966-022-01354-5

**Published:** 2022-09-17

**Authors:** Harriet Koorts, Anna Timperio, Gavin Abbott, Lauren Arundell, Nicola D. Ridgers, Ester Cerin, Helen Brown, Robin M. Daly, David W. Dunstan, Clare Hume, Mai J. M. Chinapaw, Marj Moodie, Kylie D. Hesketh, Jo Salmon

**Affiliations:** 1 Institute for Physical Activity and Nutrition (IPAN), School of Exercise and Nutrition Sciences, Deakin University, Geelong, VIC Australia; 2 Deakin University, 221 Burwood Highway, Burwood, VIC 3125 Australia; 3 Alliance for Research in Exercise, Nutrition and Activity (ARENA), Allied Health and Human Performance, University of South Australia, Adelaide, South Australia; 4 Mary McKillop Institute for Health Research, Australian Catholic University, Melbourne, Australia; 5 Centre for Sport Research, Deakin University, Geelong, Australia; 6 Baker Heart and Diabetes Institute, Melbourne, Australia; 7 School of Public Health, University of Adelaide, Adelaide, SA Australia; 8 Amsterdam UMC, Vrije Universiteit Amsterdam, Department of Public and Occupational Health, Amsterdam Public Health Research Institute, Amsterdam, the Netherlands; 9 Deakin Health Economics, Institute for Health Transformation, Deakin University, Geelong, VIC Australia

**Keywords:** Implementation, process evaluation, physical activity, sedentary behaviour, school-based intervention

## Abstract

**Background:**

*TransformUs* was a four-arm school-based intervention to increase physical activity and reduce sedentary behaviour among primary school children. Pedagogical and environmental strategies targeted the classroom, school grounds and family setting. The aims of this study were to evaluate program fidelity, dose, appropriateness, satisfaction and sustainability, and associations between implementation level and outcomes among the three intervention arms.

**Methods:**

At baseline, 18-months (mid-intervention) and 30-months (post-intervention), teachers, parents and children completed surveys, and children wore GT3X ActiGraph accelerometers for 8 days at each time point to determine physical activity and sedentary time. Implementation data were pooled across the three intervention groups and teachers were categorised by level of implementation: (i) ‘Low’ (< 33% delivered); (ii) ‘Moderate’ (33–67% delivered); and (iii) ‘High’ (> 67% delivered). Linear and logistic mixed models examined between group differences in implementation, and the association with children’s physical activity and sedentary time outcomes. Qualitative survey data were analysed thematically.

**Results:**

Among intervention recipients, 52% (*n* = 85) of teachers, 29% (*n* = 331) of parents and 92% (*n* = 407) of children completed baseline evaluation surveys. At 18-months, teachers delivered on average 70% of the key messages, 65% set active/standing homework, 30% reported delivering > 1 standing lesson/day, and 56% delivered active breaks per day. The majority of teachers (96%) made activity/sports equipment available during recess and lunch, and also used this equipment in class (81%). Fidelity and dose of key messages and active homework reduced over time, whilst fidelity of standing lessons, active breaks and equipment use increased. *TransformUs* was deemed appropriate for the school setting and positively received. Implementation level and child behavioural outcomes were not associated. Integration of *TransformUs* into existing practices, children’s enjoyment, and teachers’ awareness of program benefits all facilitated delivery and sustainability.

**Conclusions:**

This study demonstrated that intervention dose and fidelity increased over time, and that children’s enjoyment, senior school leadership and effective integration of interventions into school practices facilitated improved intervention delivery and sustainability. Teacher implementation level and child behavioural outcomes were unrelated, suggesting intervention efficacy was achieved irrespective of implementation variability. The potential translatability of *TransformUs* into practice contexts may therefore be increased. Findings have informed scale-up of *TransformUs* across Victoria, Australia.

**Trial registration:**

International Standard Randomized Controlled Trial Number ISRCTN83725066; Australian New Zealand Clinical Trials Registry Number ACTRN12609000715279. Registered 19 August 2009. Available at: https://www.anzctr.org.au/Trial/Registration/TrialReview.aspx?id=308387&isReview=true

**Supplementary Information:**

The online version contains supplementary material available at 10.1186/s12966-022-01354-5.

## Background

The positive relationships between higher levels of physical activity in children and improved cardiometabolic health risk factors, musculoskeletal health, mental health and wellbeing, cardiorespiratory fitness, and a reduced risk of unhealthy weight gain are well established [1]. More recently, time spent in prolonged sedentary behaviour (expending < 1.5 metabolic equivalents [METs] whilst in a sitting or reclining posture; e.g. computer use, TV viewing [2]) has been suggested as an independent risk factor for cardiometabolic diseases in children and youth [3–5]. However, evidence for the prospective negative association between prolonged sitting and biomedical health indicators in children remains inconclusive [6, 7]. Global physical activity guidelines recommend that children aged 5–17 years should accumulate, on average, 60 minutes of daily moderate- to vigorous-intensity physical activity (MVPA) [8]; however, the majority of children in high-income countries do not achieve this [9]. Only 22% of children from the United States (aged 6–19 years) [10] and England (aged 5-15 years) [11] achieve this guideline. In 2011–12, less than 40% of Australian children aged 9–13 years achieved the recommended 60 minutes of MVPA per day, and only 7% met the Australian sedentary behaviour guidelines of less than 2 h screen time per day [12]. Since physical activity and sedentary behaviour have been shown to track from childhood into adolescence and adulthood [13, 14], early intervention to optimise children’s physical activity and sedentary behaviour levels in childhood is preferable.

Increasingly, sedentary behaviour has been targeted in interventions alongside physical activity [15], and targeting both behaviours simultaneously may achieve the greatest health benefits [15]. Schools are ideal settings for physical activity and sedentary behaviour interventions, due to mandated schooling [16], and the majority of children spend large proportions of their waking hours sedentary at school [17, 18]. Further, making small changes to the school environment, such as installation of playground line-markings [19], equipment provision [20] and reducing playground density [21] can increase physical activity in large numbers of children, while introducing height-adjustable standing desks or classroom equipment (e.g. balls and bean bags to facilitate movement integration in the classroom) [22, 23] can reduce sedentary time. However, there remains a paucity of successful interventions combining physical activity promotion and sedentary behaviour reduction in children [24].

The lack of robust evidence for successful interventions targeting these behaviours in the school setting led to the development of a novel whole-of-school intervention to promote physical activity and reduce sedentary behaviours among 8–9 year old children, referred to as *TransformUs* [25]. The intervention incorporated behavioural and environmental strategies in the classroom, school grounds and home setting. It was based on social cognitive theory [26], behavioural choice theory [27] and ecological systems theory [28]. Alignment of these theories to the *TransformUs* intervention components and objectives is shown in Additional File 1. The efficacy of *TransformUs* was tested in a four-arm cluster-randomised controlled trial (RCT) involving 20 primary schools, 226 teachers and over 1600 children in Melbourne, Australia (2010–13). The three intervention arms targeted reductions in sedentary behaviour (SB-I group), increases in physical activity (PA-I group), and a combination of both (SB + PA-I group). These groups were compared to a usual practice control group. Six-month intervention effects of *TransformUs* showed significant increases in MVPA in the SB-I and PA-I groups during recess [29], and the SB + PA-I group spent 13.3 min/day less in weekday sedentary time compared to the control group [30]. Compared to the control group, children in the PA-I group spent 27 minutes less time sedentary at 18-months and those who received the sedentary intervention spent 5 minutes more in MVPA (at 18-months) and 33 minutes less time sedentary (at 30-months) [31].

Although the program demonstrated efficacy, the effectiveness varied by group at different time points, and the process and challenges of the implementation remain unexplored. Outcome data in isolation informs intervention effectiveness, while process evaluation determines whether an intervention is delivered or received as intended, and identifies influences on effectiveness and the potential for sustainability in routine practice [32–34]. Assessment of implementation elucidates the implications of intervention fidelity, dose and adaptation on program outcomes [35, 36]. *Fidelity* refers to the degree to which an intervention is implemented as it is prescribed in the original protocol [37], *adaptation* is the degree to which an intervention is changed or modified by a user during adoption and implementation [38], and *dose* refers to the amount of the intervention delivered [39]. Program fidelity is a particularly contested area of implementation research, as there is tension between the extent that an intervention remains ‘true’ to the program protocol to maximise the potential for positive impact, versus the reality of implementation in practice where adaptation is expected and may be encouraged for quality improvement [40]. For improved research-practice translation, there is increasing acknowledgement that interventions, and their implementation, may require ongoing adaptation for contextual relevance [41]. Yet adaptation can lead to both positive and negative outcomes on program impact. Despite that. Fidelity and adaptation ‘co-exist’ [42]. Nonetheless, the extent that adaptation impacts on program effectiveness is likely to be program, setting and population specific. The aims of this paper are therefore twofold: firstly, to assess differences in fidelity, dose, appropriateness, satisfaction and sustainability between *Transform Us!* intervention groups and over time; and secondly, to examine the associations between overall teacher implementation level (dose and fidelity across intervention groups combined) and child physical activity and sedentary behaviour outcomes. Findings from this evaluation will contribute to knowledge on effective implementation of school-based physical activity and sedentary behaviour interventions and the association between levels of implementation and intervention outcomes.

## Methods

### Overview of TransformUs

A detailed description of *TransformUs* has been published elsewhere [25], and Additional File 2 contains the TIDieR checklist. Briefly, the objectives of the intervention were to: (i) provide a whole-of-school environment that increased opportunities and support for physical activity and reducing sedentary behaviour; (ii) modify curriculum content and classroom lesson delivery to incorporate physical activity and sedentary behaviour messages, and provide an environment conducive to active behaviours; and (iii) increase awareness and opportunities for physical activity and reduced sedentary behaviours in the home setting. The underlying hypothesis was that the intervention would lead to behaviour change by impacting targeted behavioural and environmental mediators.

**Fig. 1:**
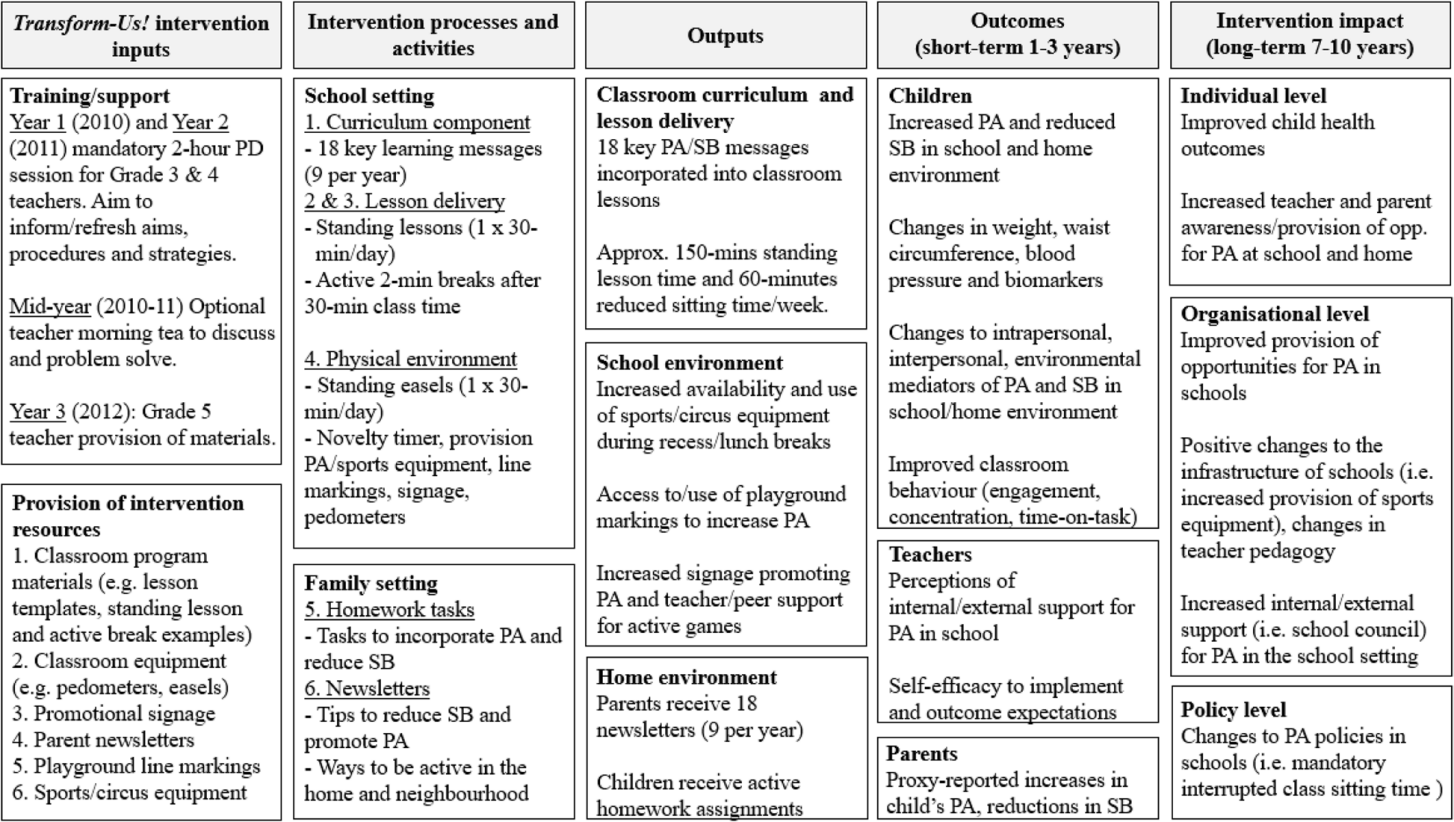
*TransformUs* Logic Model. PD: Professional Development; PA: Physical Activity; SB: Sedentary Behaviour

Complete delivery of *TransformUs* involved implementation of six behavioural and environmental components, which varied by intervention arm (SB-I, PA-I and SB + PA-I) [25]. Figure 1 presents the program logic model, Additional File 1 illustrates how the intervention components correspond to the intervention arms. The SB-I arm aimed to reduce prolonged sitting in the classroom and reduce overall sedentary time at home. The PA-I arm aimed to increase or maintain MVPA during morning recess and lunch time within school hours, and increase physical activity at home. The SB + PA-I group combined strategies from the SB and PA arms, with the aim of simultaneously reducing prolonged sedentary time and increasing physical activity at school and at home. In the first year of the intervention (2010), all Grade 3 teachers attended a 2-hour face-to-face professional development (PD) training session led by the research team. This was repeated in the second year (2011) with all Grade 4 teachers, which included teachers previously trained in Grade 3 and new teachers within Grade 4. The teacher training covered study requirements, the intervention strategies and aims of the project. A mid-year morning tea in 2011 facilitated a problem solving question and answer session. In year three (2012), when the cohort of children were in Grade 5, no face-to-face training was provided to those teachers, they only received provision of the resources. No additional implementation support was provided to teachers or schools, nor was there any ongoing implementation support provided between the data collection time points.

### Process evaluation design

A mixed method post-hoc study was adopted based on the UK Medical Research Council (MRC) recommendations for process evaluation of complex interventions [32], as it explores the unanticipated barriers and facilitators to implementation. Specifically, we used a concurrent triangulation design, whereby the qualitative and quantitative data were used in equal weighting to interpret the study findings [43]. The design was Six process indicators were included based on definitions and priority areas identified in evaluation research [44–48]. Indicators included: (i) *Fidelity* (teacher adherence to delivery of components as intended and adaptations to key message delivery); (ii) *Dose delivered* (proportion, frequency and duration of components delivered by teachers); (iii) *Dose received* (proportion, frequency and duration of components parents and children were exposed to); (iv) *Appropriateness* (teacher perceived fit, compatibility and ease of delivery of classroom components); (v) *Satisfaction* (teacher and parent planned continuation and/or support for *TransformUs*, and children’s enjoyment of the program); and (vi) *Sustainability* (facilitators and barriers to sustained implementation and integration of *TransformUs* into school policy).

### Participants

Primary schools within a 50 km radius of Melbourne, with an enrolment > 300 children and at least two Grade 3 classes, were eligible to participate in the *TransformUs* RCT [25]. Schools were stratified by low, mid and high socio-economic status (SES) based on the Australian Bureau of Statistics’ Socio-Economic Indexes for Areas (SEIFA) [49]. Of the then 1159 Government primary schools in Victoria [50], 148 (12.8%) schools were invited to participate (via fax or email) and 29 (20%) responded. A target recruitment of 20 schools (15 intervention and 5 control schools) was achieved from the 29 responders. Schools represented low (*n* = 8), mid (*n* = 11) and high (*n* = 1) SEIFA areas. Due to challenges recruiting schools representing high SES areas, the high and mid SES strata were combined for randomisation. Schools within the two SES strata were randomised by a statistician (not involved in the trial) into one of the four groups (PA-I, SB-I, SB + PA-I or control current practice [C]). All Grade 3 children (*n* = 1606) in intervention and control schools were invited to participate in the study evaluation. Grade 3 children were recruited for two reasons. Firstly, children aged 8–9 years are a target population for increases in physical activity [51], and secondly, it facilitated follow-up as children would have remained at primary school throughout the study duration [25]. Active informed parental consent was required on behalf of themselves and their child. Parents could consent for their child to take part in any combination of the evaluation assessments as involvement in all assessments was not compulsory. For the purposes of this paper, only data from children participating in the intervention were used for analyses relating to child outcomes.

### Data collection procedure

Process-level quantitative and qualitative data were collected via parent, teacher and child surveys at baseline (T1; Feb-June 2010), 6-months (T2; Nov-Dec 2010), 18-months (T3; Nov-Dec 2011) and 30-months (T4; Nov-Dec 2012). Due to low participant response rates at T2 and that the study purpose was to assess intervention implementation after substantial delivery of program components; only data collected at T1, T3 and T4 are included as part of this evaluation. Table 1 presents application of the process and outcome data against the six evaluation indicators.

**Table 1:** Application of process-level data against evaluation indicators

Process indicator & source	Assessment criteria	Source, time point and number of survey items	Example survey item
**Fidelity**	Teacher adherence to delivery of *TransformUs* as intended, and adaptation of key messages.	Teacher survey (T3 & T4 9 items) Teacher lesson evaluations (Years one & two, 1 item)	‘*Is sports equipment freely available to all students at recess/lunch?*’
	Facilitators and barriers to intervention delivery (qualitative)	Teacher survey (T3, 9 items; T4, 13 items) Teacher lesson evaluations (Years one & two, 1 item)	‘*If you did not deliver all key messages/lessons, why?*’
**Dose delivered**	Proportion and frequency of *TransformUs* components delivered by teachers	Teacher survey (T3, 5 items; T4, 10 items)	‘*How long were children standing for during each standing lesson?*’
**Dose received**	Proportion and frequency of *TransformUs* components received by parents and children	Child survey (T3, 12 items; T4, 11 items)	‘*How many TransformUs newsletters did you receive and take home?*’
		Parent survey (T3, 3 items; T4, 1 item)	‘*Did your child’s teacher set your child active/standing homework?*
**Appropriateness**	Perceived fit, compatibility and ease of delivery of *TransformUs* classroom components	Teacher survey (T3 & T4 13 items)	‘*Is it feasible to integrate standing lessons in classrooms?*’
**Satisfaction**	Enjoyment or approval to continue delivering or receiving *TransformUs*	Teacher survey (T3, 2 items; T4, 1 item)	‘*Would you recommend TransformUs to other teachers?’*
		Child survey (T3, 7 items; T4, 8 items)	*‘How much did you like standing during class lessons?*’
		Parent survey (T3, 1 items; T4, 2 items)	‘*Would you like your child to continue TransformUs strategies?*’
**Sustainability**	Facilitators and barriers to sustained implementation and integration of *TransformUs* into school policy (qualitative)	Teacher survey (T3, 8 items; T4, 4 items)	‘*Will you continue with the TransformUs strategies? Why/why not?*’

T3: 18-months (Nov-Dec 2011). T4: 30-months (Nov-Dec 2012). Assessment criteria (Column 2) refers to quantitative data unless otherwise specified

### Measures

Baseline evaluation surveys captured teacher, parent and child socio-demographic characteristics.

#### Process evaluation indicators

Quantitative data provided information on environmental context (school characteristics), teacher delivery context (teaching grades), and attendance at *TransformUs* training. Surveys at T3 and T4 assessed teacher fidelity, dose delivered, appropriateness, satisfaction and sustainability, and parent and child satisfaction and dose received (example survey items in Table 1 and an example teacher survey is shown in Additional File 3). Lesson evaluations in year one (2010) and year two (2011) were used to assess teacher fidelity and adaptations to the key messages. Each key message included three components: a class discussion, class activity and individual child worksheet. For each of the nine key messages, teachers were asked to record in lesson evaluations any barriers and facilitators to implementation (free text response), and how they delivered the key messages (response options: ‘I used the materials as they are’, ‘I modified the materials’ or ‘I created new materials’). Fidelity to the intervention protocol was assessed based on the extent that teachers reported delivery of the key messages using the ‘materials as they are’. Qualitative open-ended survey responses contributed to assessing barriers and facilitators to intervention adoption, delivery, sustainability and integration into school policy (example survey items in Table 1). Survey questions were refined between the T3 and T4 data collections to improve comprehension and reduce participant burden. Some questions were reworded or removed as a result.

#### Physical activity, sedentary time and sedentary breaks

Children wore a hip-mounted ActiGraph GT3X (Pensacola, FL) accelerometer for 8 consecutive days during waking hours (excluding water-based activities) to obtain behaviour data. Time (mins) spent in sedentary, light-intensity physical activity (LPA) and MVPA, and frequency of breaks in sedentary time, on an average weekday, average weekend day and average day were calculated. Accelerometry data were considered valid if there was a minimum of 4 days, including one weekend day, with at least 8 h per day of wear time (or at least 50% wear time within periods of the day i.e. class time) [52]. A 15-second epoch length was used and sedentary time was defined as < 25 counts per 15-second epoch [53]. The number of breaks in sedentary time was defined as the frequency of occasions that the accelerometer data exceeded 100 counts.min-1 [54]. The Freedson age-adjusted cutpoints [55] were used to calculate time spent in light- (1.5–3.9 METs), moderate- (4.0–5.9 METs) and vigorous-intensity (≥6.0 METs) physical activity. Average wear time for valid periods and valid days were calculated and non-wear time was defined as a period of ≥20 minutes of consecutive zeros [56]. A detailed description of the accelerometry measurement protocol and data management has been previously published [25].

### Data analyses

As the study purpose was to evaluate process-related factors amongst participants delivering or receiving *TransformUs*, no control group comparisons were made. Comparisons between intervention groups (groups PA-I, SB-I and SB + PA-I) were examined separately at T3 and T4, using linear mixed-effects models for continuous variables and logistic mixed-effects models for dichotomous variables, with random intercepts for school, and adjusted for school-level SES (mid/high vs. low). In the models, study intervention arm was the independent variable and the different process evaluation indicators (i.e., dose delivered) were the dependent variables. Due to small cell sizes for binary teacher outcomes, inferential models were not conducted, while group comparisons of continuous teacher outcomes were made using unadjusted linear regression models. Bias-corrected bootstrapping (1000 resamples) was used to produce robust standard errors. An implementation score for each teacher was generated by summing their scores for fidelity and dose delivered, corresponding to the number of intervention components within each intervention group. The maximum score attainable (indicating complete program delivery), varied by group: PA-I (max score 6), SB-I (max score 4) and SB + PA-I (max score 8). For data to be considered valid, responses to a minimum of 5 of the 6 components was required for the PA-I group and a minimum of 7 of the 8 items for the SB + PA-I group. Due to the small number of intervention components in the SB-I group (*n* = 4), complete data for all 4 components was required. In instances of missing data for one component in the PA-I and SB + PA-I groups, the mean score of the remaining components was imputed. At T3, 46 of the 60 teachers (77%) and at T4, 28 of the 92 teachers (30%) provided valid data.

Teachers were grouped by level of implementation based on the proportion of the entire intervention delivered (dose delivered and fidelity). Consistent with previous research [57, 58], implementation levels corresponded to: (i) ‘low’ (< 33% of the entire intervention delivered); (ii) ‘moderate’ (33–67% delivered); and (iii) ‘high’ (> 67% delivered). To examine associations between teacher implementation level and child behavioural outcomes, linear mixed models with random intercepts for school were fitted. Due to small samples, teacher implementation data were pooled across intervention groups, to calculate a standardised mean implementation score. Analyses were adjusted for potential confounders of school socioeconomic position (SEP), average accelerometry wear time, baseline values of the outcome variables and intervention group. In the models where sedentary breaks were included as the outcome variable, analyses were also adjusted for average sedentary time. All statistical analyses were conducted using Stata (SEv17) and statistical significance was set at *p* < 0.05.

Qualitative survey data, from open-ended survey responses and lesson evaluations, were entered into NVivo10 and analysed thematically. Thematic analysis was conducted by HK, who has expertise in qualitative analysis, and involved preliminary data familiarisation, coding and tabulation of raw themes. Raw themes were then grouped into major themes based on patterns of emergence and overlapping relevance. Coding and theme development was initially deductive, based on the study aims and underlying theory, followed by an inductive process directed by the content of the data [59]. Barriers and facilitators were grouped according to their level of impact (organisational or individual).

## Results

Across the 15 intervention schools recruited, 1134 children were eligible to take part in the trial. Of these, 443 (39%) provided consent to participate in at least one evaluation assessment. Survey and valid accelerometry data were obtained from 253 children in the PA-I (*n* = 97), SB-I (*n* = 81) and SB + PA-I (*n* = 75) groups, and survey consent was obtained from 163 teachers and 1141 parents. Participant response rates at T3 and T4 are shown in an a (see Additional File 4).

### Sample characteristics

Baseline surveys were completed by 52% (*n* = 85) of teachers, 92% (*n* = 407) of children and 29% (*n* = 331) of parents in the intervention groups. The mean (SD) age of teachers was 37 (12.5) years, the majority were female (83%) and employed full time (93%), and on average had taught for 12.4 (12.1) years. The mean age of children was 8.2 (0.5) years and girls represented 58% of the sample. Most parents (88%) who completed a survey were female and the mean age was 39.4 (5.1) years. Among responding teachers at T3, 20% reported having attended *TransformUs* training in 2010, 30% in 2011, and 25% attended the mid-morning (problem-solving) tea in 2011.

### Fidelity and dose delivered (Teachers)

#### Key messages and active/standing homework (PA-I, SB-I, SB + PA-I)

Tables 2 and 3 present teacher fidelity and dose delivered by intervention group. Responding teachers delivered approximately 70% of the nine key messages at T3 (M = 6.3, SD = 2.9), reducing to 48% by T4 (M = 4.4, SD = 3.5). The dose of key message delivery was statistically significantly greater in the PA-I group compared to the SB-I and SB + PA-I group at T3 and T4 (Table 3). Only 31% of teachers delivered all nine key messages at T3, which reduced over time, and very few teachers delivered all nine key messages without any adaptation in either 2010 (15%) or 2011 (9%) (i.e., delivered one or two rather than all three key message components) (Table 2). Fidelity of key message delivery was low across all intervention groups, although consistently higher in the PA-I group; significant at T3. The majority (65%) of teachers set active/standing homework at T3 (fidelity). Homework in general was set once or more per week by the majority (54%) of teachers, whereas only 19% set active/standing homework once or more per week. Fidelity to delivery of both components declined over time. The frequency (dose) of active/standing homework delivery was low across all groups, although was consistently higher in the PA-I group over time (Table 3).

**Table 2:** Teacher intervention fidelity and dose delivered (dichotomous outcomes) at T3 and T4

Intervention component	***T3***					***T4***				
	N	Yes (%)	Intervention group			N	Yes (%)	Intervention group		
			PA-I	SB-I	SB + PA-I			PA-I	SB-I	SB + PA-I
			Yes (%)	Yes (%)	Yes (%)			Yes (%)	Yes (%)	Yes (%)
***Teacher fidelity***			**Max** ***N*** **= 24**	**Max** ***N*** **= 13**	**Max** ***N*** **= 24**			**Max** ***N*** **= 41**	**Max** ***N*** **= 14**	**Max** ***N*** **= 23**
Delivered all nine key messages?	59	31	41	31	21	53	26	33	0	25
Delivered all nine key messages without adaptation?	47	15	8	11	29	35	9	7	29	0
Delivered one standing lesson p/day?	33	30	–	17	38	36	56	–	64	50
Children completed active breaks?	34	56	–	25	73	35	66	–	83	57
Sports equipment was available during recess/lunch?	45	96	96	–	96	48	58	76	–	96
Used sports equipment during class time?	42	81	65	–	96	46	89	88	–	91
Signage promoted physical activity during recess/lunch?	44	91	91	–	91	51	43	33	–	57
Used line markings during class time?	42	60	60	–	59	54	72	69	–	77
Set active/standing homework?	46	65	82	80	42	66	39	49	42	24
***Teacher dose delivered***			**Max** ***N*** **= 22**	**Max** ***N*** **= 13**	**Max** ***N*** **= 24**			**Max** ***N*** **= 48**	**Max** ***N*** **= 14**	**Max** ***N*** **= 27**
Used sports equipment during class time ≥ once/wk.?	36	58	40	–	71	42	67	62	–	71
Used line markings during class time ≥ once/wk.?	27	52	39	–	64	41	54	50	–	58
Active/standing homework delivered ≥once/wk.?	57	19	30	21	9	89	15	10	21	19

Total N and N Yes = Intervention groups combined. *PA-I* Physical activity intervention group, *SB-I* Sedentary behaviour intervention group, SB + PA-I=Combined physical activity and sedentary behaviour group. T3: 18-months (Nov-Dec 2011). T4: 30-months (Nov-Dec 2012). Inferential analyses were not conducted between groups due to small cell sizes

^a^Data relates to teacher weekly lesson evaluations at 2010 and 2011

**Table 3:** Teacher between-group comparison of teacher intervention dose delivered (continuous outcomes) at T3 and T4

Intervention component	N	Intervention group						Group effect *p*-value	Mean differences^a^		
		PA-I		SB-I		SB + PA-I			SB-I vs PA-I	SB + PA-I vs PA-I	SB + PA-I vs SB-I
		M (SD)	Med (IQR)	M (SD)	Med (IQR)	M (SD)	Med (IQR)		B (95% CI)	B (95% CI)	B (95% CI)
***Teacher dose delivered T3***		**Max** ***N*** **= 22**		**Max** ***N*** **= 13**		**Max** ***N*** **= 24**					
No. key messages delivered (n)	59	7.6 (1.5)	8 (5–9)	5.4 (3.8)	8 (0–9)	5.8 (2.9)	6.5 (0–9)	0.007	−2.16 (−4.31, −0.01)	−1.80 (−3.09, −0.50)	0.37 (−2.00, 2.73)
Active Breaks duration (mins)	15	–	–	2.7 (0.6)	3 (2–3)	4.8 (3)	3.5 (2–10)	0.012	–	–	2.17 (0.47, 3.87)
***Teacher dose delivered T4***	**N**	**Max** ***N*** **= 30**		**Max** ***N*** **= 14**		**Max** ***N*** **= 23**			**SB–I vs PA–I**	**SB + PA–I vs PA–I**	**SB + PA-I vs SB-I**
No. key messages delivered (n)	53	4.9 (3.6)	4.5 (0–9)	2.1 (2.3)	2 (0–5)	4.4 (3.7)	4.5 (0–9)	0.038	−2.72 (−4.85, −0.60)	−0.49 (− 2.71, 1.73)	2.23 (− 0.24, 4.71)
Days/week delivered standing lesson (n)	20	–	–	1.7 (1.3)	1 (1–5)	2.7 (1.3)	3 (1–5)	0.065	–	–	1.06 (−0.07, 2.19)
Times/day delivered standing lesson (n)	20	–	–	1.6 (0.5)	2 (1–2)	1.1 (0.5)	1 (0–2)	0.018	–	–	−0.54 (−0.99, − 0.09)
Standing lesson duration (mins)	21	–	–	17.3 (11.6)	17 (5–40)	13.7 (5.5)	11 (7–25)	0.37	–	–	−3.67 (−11.69, 4.36)
Days/week delivered an active break (n)	23	–	–	3.7 (1.3)	3.5 (1–5)	3.2 (1.5)	3 (1–5)	0.37	–	–	−0.55 (−1.75, 0.66)
Times/day delivered an active break (n)	22	–	–	2.2 (1.2)	2 (1–5)	2.4 (1.3)	2 (1–6)	0.75	–	–	0.16 (−0.84, 1.16)
Active break duration (mins)	21	–	–	3 (1.2)	3 (1–5)	5.2 (5.4)	3 (1–20)	0.14	–	–	2.15 (−0.74, 5.04)

Total N=Intervention groups combined

^a^Bootstrapped linear regression models

*PA-I* Physical activity intervention group, *SB-I*Sedentary behaviour intervention group, SB + PA-I=Combined physical activity and sedentary behaviour group. T3: 18-months (Nov-Dec 2011). T4: 30-months (Nov-Dec 2012). Empty cells relate to questions not being asked of that group due to lack of relevance

#### Standing lessons and active breaks (SB-I, SB + PA-I)

At T3, 30% of teachers delivered one standing lesson per day and 56% delivered active breaks (Table 2). Teacher fidelity to standing lesson and active break delivery increased over time, remaining consistently higher in the SB + PA-I group. The dose of active break delivery was consistent across both groups at T4; however, the teacher reported duration was significantly greater in the SB + PA-I group, compared to the SB-I group, at T3 (Table 3). The dose of standing lesson and active break delivery was not measured at T3.

#### Physical activity/sports equipment, line markings and promotional signage use (PA-I, SB + PA-I)

The frequency (dose) of weekly physical activity/sports equipment, signage and line marking use was consistent across the PA-I and SB + PA-I groups (Table 2). Almost all teachers made physical activity/sports equipment available during recess and lunch, and used physical activity/sports equipment in class. Over half of all teachers reported using physical activity/sports equipment in class once or more per week, and this increased over time (Table 2). Teacher fidelity for physical activity/sports equipment use in class remained consistently greater in the SB + PA-I group over time (Table 2). Teachers were only encouraged to use line markings during recess and lunch, yet reported using line markings during class time and this increased over time. Over half of all teachers consistently used line markings once or more per week in class, particularly in the SB + PA-I group (Table 2). Teacher fidelity to promotional signage use reduced by over half of all teachers between T3 and T4.

### Fidelity and dose delivered (Teachers Qualitative data)

Qualitative data from open-ended questions in teacher surveys and lesson evaluations captured facilitators and barriers to intervention delivery and adherence (dose and fidelity) (Table 4). Themes were broadly consistent at T3 and T4 and across intervention groups, although response rates to the open-ended questions were low amongst all intervention groups at both time points.

**Table 4:** Barriers and facilitators to teacher intervention delivery, sustained implementation and integration into school policy

Level of impact	Facilitators *N* = 60 teachers (T3), *N* = 92 teachers (T4)	Barriers *N* = 60 teachers (T3), *N* = 92 teachers (T4)
**Intervention delivery**		
*Organisational*	Integration into and expansion of existing practices	Lack of awareness participating/program promotion
	Supporting school ethos and infrastructure	Crowded curriculum
		Practicalities/setting characteristics (i.e. classroom size)
		^**a**^No homework policy
		^**a**^Parental lack of support for active/standing homework
*Individual*	Children’s enjoyment	Lack of time
	Teacher awareness and understanding of values/benefits	Associated with disruptions or distractions
	Freedom to incorporate when required	Forgetting to implement
		Perceived appropriateness (i.e. behavioural difficulties)
		Perceived lack of benefits or value
		^**a**^Lack of awareness/planning
**Sustained intervention implementation**		
*Organisational*	Integrates into existing teaching practices	Time
	Integrates into other school areas	Insufficient integration of key messages across curriculum
	Regular professional development, implementation support	Lack of consistent reinforcement/awareness of program
	School leadership and support	
	Raising profile of physical activity as a priority in the school	
*Individual*	Awareness of program benefits to teaching	Perceptions of program impact
	Awareness of program benefits among children	Demands of complete program delivery
	Children’s enjoyment	Perception of work and integration into existing practices
	Increased ideas and program materials	
**Intervention integration into school policy**		
*Organisational*	Integration and prioritisation in school/curriculum planning	Mandating the program as a policy unsupported by school
	Facilitate integration into existing curriculum	Practicalities (i.e. classroom infrastructure)
	Incorporate as part of teacher training/PD sessions	Gaining whole-of-school and committee support
	Whole of school and leadership support	Time and crowded curriculum
	Prioritising program within a supportive planning strategy	
*Individual*	Increase awareness and promotion of values/benefits	Perceived value of program components
	Reinforce teacher commitment and support for delivery	

Themes are ranked in order of frequency of emergence. Total N represents number of responding intervention group teachers. T3: 18-months (Nov-Dec 2011). T4: 30-months (Nov-Dec 2012)

^**a**^Theme relates only to intervention component active/standing homework. Data from open-ended qualitative survey responses

#### Facilitators to program delivery

At T3, the most frequently reported theme was children’s enjoyment of *TransformUs* as a facilitator to implementation. Teachers described children’s pleasure in participating in the program’s physically active elements and the children’s ability to refocus quickly after active breaks. This included an awareness and understanding of program values and benefits as influencing their decision to integrate the program:

“*Has many benefits and makes the classroom much more fun and I think, even more relaxed!*” [T3, Grade 3/4 Teacher, SB + PA-I]

Implementation was also supported by the teachers’ freedom and flexibility to deliver aspects of the program when required, although identified only at T4. At an organisational level, successful integration of the program into, and expansion of, existing teaching practices was the most frequently reported enabling factor for implementation. One teacher referred to a supporting school ethos and infrastructure to facilitate program implementation, and one other described the program as expanding their existing teaching practices:

“*The concept of more active participation, lessons, standing, etc has been successfully integrated*. *TransformUs made us think beyond what we were already doing*” [T4, Grade 3/4 Teacher, PA-I]

#### Barriers to program delivery

At both time points, the most consistently reported barrier to program delivery at an individual level was time constraints. Teachers referred to competing demands and conflicts between timetable content and delivery. Some teachers associated active breaks and standing lessons with disruptions or distractions to the class. Specifically, these were described in reference to differences in children’s learning styles and classroom behaviour, and the potential inappropriateness of these components among children with behavioural difficulties or additional learning needs. The third most commonly reported theme by teachers, was forgetting to implement. This was reported as a barrier to key message, standing lesson and active break delivery:

*“At times we forgot to plan this* [standing lessons] *into our program”* [T3, Grade 3/4Teacher, SB + PA-I]

Organisational level barriers included the absence of ‘top-down’ school promotion, resulting in some teachers lacking an awareness of participation, in particular at T4. This was attributed to insufficient leadership and promotion of the program at the school level, including weaknesses in communication leading to program discontinuation. A ‘crowded curriculum’ was the second most common theme, followed by classroom infrastructure (such as room size), which hindered successful delivery of standing lessons for some teachers. At both time points, a small number of teachers reported either having a ‘no homework’ policy or identified a lack of parental support as a barrier to active/standing homework delivery:

“*Parents didn’t want it* [active/standing homework]*, they wanted homework that develops skills in coming home, planning time sitting and concentrating, as this will prepare them for high school etc*.” [T3, Grade 4 Teacher, SB + PA-I]

### Dose received (Parents and Children)

#### Parents

##### Key messages and newsletters (PA-I, SB-I, SB + PA-I)

Tables 5 and 6 present dose received by parents and children. At T3, parents reported receiving on average 3.42 (SD 1.9) of the nine newsletters and reported trialling an average 2.27 (SD 1.9) of the nine key messages at home. Parents in the SB-I group received a significantly greater number of newsletters compared to the PA-I group at T3 (Table 6). At T4, 62% of parents reported their child mentioning more than one key message at home (Table 5).

**Table 5:** Parent and child dose received (dichotomous outcomes) at T3 and T4

Intervention component	N	Yes (%)	Intervention group			Group effect *p*-value	Adjusted Odds Ratios^a^		
			PA-I	SB-I	SB + PA-I		SB-I vs PA-I	SB + PA-I vs PA-I	SB + PA-I vs SB-I
			Yes (%)	Yes (%)	Yes (%)		OR (95% CI)	OR (95% CI)	OR (95% CI)
***Parents dose received T3***			**Tot** ***N*** **= 102**	**Tot** ***N*** **= 90**	**Tot** ***N*** **= 87**				
Received any newsletters?	279	77.1	80.4	80	70.1	0.11	1.04 (0.44, 2.47)	0.48 (0.20, 1.11)	0.46 (0.20, 1.05)
***Parents dose received T4***			**Tot** ***N*** **= 87**	**Tot** ***N*** **= 59**	**Tot** ***N*** **= 65**				
Child mentioned ≥1 key message?	211	61.6	71.3	55.9	53.8	0.049	0.45 (0.21, 0.97)	0.44 (0.20, 0.93)	0.97 (0.44, 2.16)
***Children dose received T3***			**Max** ***N*** **= 159**	**Max** ***N*** **= 117**	**Max** ***N*** **= 132**		**SB-I vs PA-I**	**SB + PA-I vs SB-I**	**SB + PA-I vs PA-I**
Lesson involved standing/moving ≥once/week?	249	64.7	–	52.1	75.8	< 0.0005	–	–	3.50 (1.81, 6.75)
Class had standing breaks after sitting a long time ≥ once/week?	247	65.6	**–**	59	71.5	0.056	**–**	**–**	1.86 (0.98, 3.51)
Teacher sometimes/always ensures not sitting a long time?	249	72.7	**–**	71.8	73.5	0.77	**–**	**–**	1.10 (0.58, 2.07)
Teacher sometimes/always does lots of class activities standing?	249	50.6	**–**	44.4	56.1	0.069	**–**	**–**	1.66 (0.96, 2.86)
Teacher sometimes/always ensures move a lot during class?	247	61.5	**–**	62.1	61.1	0.91	**–**	**–**	0.97 (0.54, 1.74)
^b^Allowed to use sports equipment during recess/lunch?	289	97.2	96.8	–	97.7	b	–	–	–
More signs in yard promoting activity?	243	35.8	32.6	**–**	39.6	0.32	–	1.35 (0.75, 2.41)	–
Teachers encouraged physical activity?	243	55.1	54.6	**–**	55.9	0.81	–	1.07 (0.61, 1.88)	–
Teacher set active/standing homework?	375	51.5	69.8	34.9	40	<.0005	0.20 (0.10, 0.39)	0.25 (0.10, 0.39)	1.26 (0.66, 2.41)
Completed active/standing homework?	293	64.8	74.1	52.7	59.2	0.014	0.41 (0.20, 0.82)	0.38 (0.18, 0.80)	0.93 (0.44, 1.94)
***Children dose received T4***			**Max** ***N*** **= 139**	**Max** ***N*** **= 92**	**Max** ***N*** **= 102**		**SB-I vs PA-I**	**SB + PA-I vs SB-I**	**SB + PA-I vs PA-I**
Lessons involved standing/moving ≥once/week?	192	63.5	–	65.9	61.4	0.60	–	–	0.84 (0.44, 1.62)
Class had standing break after sitting ≥once/week?	193	58	–	55.4	60.4	0.59	–	–	1.17 (0.66, 2.11)
Teacher sometimes/always ensures not sitting a long time?	194	65.5	–	63	67.7	0.45	–	–	1.28 (0.67, 2.43)
Teacher sometimes/always does lots of class activities standing?	194	39.7	–	34.8	44.1	0.21	–	–	1.48 (0.80, 2.73)
Teacher sometimes/always ensures move a lot during class?	194	47.4	–	44.6	50	0.46	–	–	1.26 (0.68, 2.33)
^b^Allowed to use sports equipment during recess/lunch?	241	93.8	93.5	–	94.1	b	–	–	–
Noticed little/lot more active signage in school yard?	155	40.6	41.2	–	39.7	0.83	–	0.92 (0.45, 1.90)	–
Teachers gave little/lot more encouragement to be active?	155	47.7	48.5	–	46.6	0.81	–	0.92 (0.46, 1.83)	–
Teacher set active/standing homework?	333	27.3	36.7	21.7	19.6	0.013	0.50 (0.25, 0.99)	0.38 (0.19, 0.76)	0.76 (0.34, 1.66)
Completed active/standing homework?	131	65.6	70.4	60.7	59.4	0.56	0.63 (0.22, 1.84)	0.60 (0.21, 1.75)	0.95 (0.29, 3.14)

Total N and N Yes = Intervention groups combined. *PA-I* Physical activity intervention group, *SB-I* Sedentary behaviour intervention group, SB + PA-I=Combined physical activity and sedentary behaviour group. T3: 18-months (Nov-Dec 2011). T4: 30-months (Nov-Dec 2012)

^a^Bootstrapped logistic mixed models adjusted for school SES

^b^Inferential analysis not conducted due to insufficient cell sizes. Empty cells relate to questions not being asked of that group due to lack of relevance

**Table 6:** Parent and child between-group comparison of intervention dose received (continuous outcomes) at T3 and T4

Intervention component	N	Intervention group						Group effect *p*-value	Adjusted mean differences^a^		
		PA-I		SB-I		SB + PA-I			SB-I vs PA-I	SB + PA-I vs PA-I	SB + PA-I vs SB-I
		M (SD)	Med (IQR)	M (SD)	Med (IQR)	M (SD)	Med (IQR)		B (95% CI)	B (95% CI)	B (95% CI)
***Parents dose received T3***		**Max** ***N*** **= 102**		**Max** ***N*** **= 90**		**Max** ***N*** **= 87**					
Key messages tried (n)	119	2.6 (1.9)	2 (1–8)	2.1 (1.5)	2 (1–8)	2.0 (1.6)	1 (0–8)	0.31	−0.41 (−1.11, 0.30)	−0.55 (−1.30, 0.20)	−0.15 (−0.87, 0.58)
Newsletters received (n)	154	3.1 (2)	3 (1–10)	4 (1.9)	4 (0–8)	3.4 (1.8)	3.5 (1–6)	0.038	0.97 (0.23, 1.71)	0.38 (−0.31, 1.08)	−0.58 (−1.33, 0.16)
***Children dose received T3***	**N**	**Max** ***N*** **= 159**		**Max** ***N*** **= 117**		**Max** ***N*** **= 132**					
Key messages recalled (n)	407	5.6 (2.2)	6 (0–9)	4.6 (2.6)	5 (0–9)	4.4 (2.7)	5 (0–9)	0.008	−0.81 (− 1.40, − 0.22)	−0.83 (− 1.45, − 0.21)	− 0.02 (− 0.65, 0.61)
^b^Newsletters received (n)	402	2.9 (1.2)	3 (1–5)	3.2 (1.4)	3 (1–5)	2.8 (1.3)	3 (1–5)	0.040	0.49 (−0.12, 1.09)	−0.30 (− 0.87, 0.27)	−0.79 (− 1.40, 0.18)
***Children dose received T4***	**N**	**Tot** ***N*** **= 139**		**Tot** ***N*** **= 92**		**Tot** ***N*** **= 102**					
Key messages recalled (n)	333	4.4 (2.9)	4 (0–10)	3.6 (2.9)	3 (0–10)	3.4 (3)	3 (0–10)	0.017	−0.77 (−1.54, 0.00)	−1.00 (− 1.74, − 0.27)	−0.23 (− 1.05, 0.58)

Total N=Intervention groups combined.*PA-I* Physical activity intervention group, *SB-I* Sedentary behaviour intervention group, SB + PA-I=Combined physical activity and sedentary behaviour group

^a^Estimated mean differences from bootstrapped linear mixed models adjusted for school SES. T3: 18-months (Nov-Dec 2011). T4: 30-months (Nov-Dec 2012)

^b^Response options were 1 = none, 2 = 1–2 newsletters, 3 = 3–4 newsletters, 4 = 5–6 newsletters, 5 = 7–9 newsletters

#### Children

##### Key messages (PA-I, SB-I, SB + PA-I)

Children in the PA-I group recalled being taught significantly more key messages than any other group at T3 and T4 (Table 6).

##### Standing lessons and active breaks (SB-I, SB + PA-I)

More than half of all children at both time points received a standing lesson at least once per week, and this was significantly greater in the SB + PA-I compared with the SB-I group at T3 (Table 5). Consistent across intervention groups, children’s reported exposure to teacher strategies to reduce sitting was consistently greater at T3 than T4. Some 73% of children at T3 reported that their teacher ‘sometimes’ or ‘always’ did not let them sit down for too long, and over half reported doing ‘lots of class activities standing up’ and that the teacher gets them to ‘move around a lot in class’ (Table 5). Most children (66%) reported having an active break at least once per week at T3, which reduced over time.

##### Encouragement of physical activity, promotional signage and physical activity/sports equipment (PA-I, SB + PA-I)

Consistent across intervention groups, 55% of the children noticed that their teacher encouraged physical activity at T3, reducing to 48% at T4. Thirty-six percent of children noticed more signs promoting physical activity in the school grounds at T3 and this increased to 41% at T4 (Table 5). Almost all children at both time points reported being allowed to use physical activity/sports equipment during recess and lunch breaks (Table 5).

##### Active/standing homework (PA-I, SB-I, SB + PA-I)

Active homework was reported by half of all children a T3. This proportion decreased at T4, but was significantly higher in the PA-I group, compared to the other two groups, at both time points (Table 5). At T3, the PA-I group also had higher odds of completing the active homework than the other two groups.

### Appropriateness (Teachers)

Table 7 presents teacher perceptions of the appropriateness of the program. Overall, the program was reported as appropriate for the classroom setting. At T3, the majority of teachers reported that low amounts of preparation were required to implement the key messages (76%), standing lessons (72%), active breaks (82%) and active/standing homework (88%). The key messages, standing lessons and active breaks were perceived as easy to deliver. The proportion of teachers reporting high levels of appropriateness for standing lessons (amount of preparation required for delivery, ease of implementation, feasible integration into their current learning theme and feasible integration into the classroom) increased over time. The proportion of teachers reporting high levels of appropriateness for active breaks also increased over time (Table 7). The item scoring least favourably was integration of the key messages into the current learning theme. At T3, only 34% of teachers reported that key messages could be easily integrated, which was consistent across all intervention groups.

**Table 7:** Teacher-reported appropriateness and satisfaction with *TransformUs* components (dichotomous outcomes) at T3 and T4

Intervention component	***T3***					***T4***				
	N	Yes (%)	Intervention group			N	Yes (%)	Intervention group		
			PA-I	SB-I	SB + PA-I			PA-I	SB-I	SB + PA-I
			Yes (%)	Yes (%)	Yes (%)			Yes (%)	Yes (%)	Yes (%)
***Teacher perceived appropriateness***			**Max** ***N*** **= 22**	**Max** ***N*** **= 14**	**Max** ***N*** **= 21**			**Max** ***N*** **= 40**	**Max** ***N*** **= 12**	**Max** ***N*** **= 21**
Key messages required low preparation?	53	76	82	73	70	45	64	50	100	65
Key messages were highly easy to deliver?	52	85	86	91	80	45	80	80	100	71
Key messages highly integrated into learning theme?	53	34	32	18	45	45	60	70	25	65
Standing lessons required low preparation?	32	72	–	73	71	22	91	–	91	91
Standing lessons were highly easy to deliver?	32	78	–	82	76	21	95	–	100	91
Standing lessons highly integrated into learning theme?	32	50	–	55	48	21	71	–	80	64
Standing lessons were feasibly integrated in classrooms?	31	52	–	67	42	33	79	–	75	90
Active breaks required low preparation?	28	82	–	100	76	23	87	–	100	77
Active breaks were highly easy to deliver?	28	79	–	86	76	23	78	–	90	69
Active breaks highly integrated into learning theme?	28	61	–	29	71	23	78	–	60	92
Active breaks were feasibly integrated in classrooms?	29	69	–	60	74	31	74	–	60	81
Active/standing homework required low preparation?	34	88	90	–	86	22	73	75	–	67
Active/standing homework highly integrated into learning theme?	34	44	50	–	36	22	64	63	–	67
***Teacher satisfaction***			**Max** ***N*** **= 22**	**Max** ***N*** **= 14**	**Max** ***N*** **= 21**			***N*** **= 47**	***N*** **= 12**	***N*** **= 25**
Would recommend *TransformUs* to other teachers?	56	77	77	67	82	84	81	72	92	92
^a^Will continue *TransformUs* strategies?	55	86	77	91	91	–	–	–	–	–

Total N and N Yes = Intervention groups combined. *PA-I* Physical activity intervention group, *SB-I* Sedentary behaviour intervention group, SB + PA-I=Combined physical activity and sedentary behaviour group. T3: 18-months (Nov-Dec 2011). T4: 30-months (Nov-Dec 2012). ^a^Question not asked either at T3 or T4. Empty cells relate to questions not being asked of that group due to lack of relevance

### Satisfaction (Teachers, Parents and Children)

Table 7 also presents teacher reported satisfaction with the program, and Table 8 presents parent and child reported satisfaction with the program. *TransformUs* was positively received by teachers, parents and children. In terms of teacher perceived impact of the program, at T3 and T4, teachers perceived that children had greater concentration after an active break (71 and 85%, respectively), and after a standing lesson (52 and 78%, respectively). Teachers also perceived that children had greater time-on-task after an active break (68% at T3; 82% at T4), and after a standing lesson (48% at T3; 79% at T4). At T3, 86% of teachers planned to continue *TransformUs* strategies and 77% stated they would recommend *TransformUs* to other teachers (Table 7). The majority of parents supported their child’s continued participation in the program, which increased over time, and 69% perceived that *TransformUs* benefitted their child’s learning at T4 (Table 8). At both time points, the majority of children enjoyed standing lessons, active breaks and active/standing homework, indicating that they would like more standing lessons and active breaks in the future (Table 8). At T3, a quarter of the children reported that it was easier to listen to the teacher/complete work when standing, and over half reported it was easier to listen/complete work after an active break (Table 8).

**Table 8:** Parent- and child-reported satisfaction with *TransformUs* components (dichotomous outcomes) at T3 and T4

Intervention component	N	Yes (%)	Intervention group			Group effect *p*-value	Adjusted Odds Ratios^a^		
			PA-I	SB-I	SB + PA-I		SB-I vs PA-I	SB + PA-I vs PA-I	SB + PA-I vs SB-I
			Yes (%)	Yes (%)	Yes (%)		OR (95% CI)	OR (95% CI)	OR (95% CI)
***Parent satisfaction T3***			**Tot** ***N*** **= 93**	**Tot** ***N*** **= 85**	**Tot** ***N*** **= 85**				
Would like child to continue *TransformUs*?	263	71.1	79.6	69.4	63.5	0.092	0.57 (0.27, 1.23)	0.45 (0.21, 0.93)	0.78 (0.37, 1.61)
***Parent satisfaction T4***			**Max** ***N*** **= 86**	**Max** ***N*** **= 59**	**Max** ***N*** **= 68**				
Would like child to continue *TransformUs*?	213	79.3	86	74.6	75	0.28	0.49 (0.19, 1.31)	0.50 (0.19, 1.30)	1.02 (0.42, 2.47)
^a^*TransformUs* strategies benefited child’s learning?	202	68.8	74.4	67.9	62.5	0.34	0.72 (0.31, 1.66)	0.57 (0.26, 1.22)	0.79 (0.34, 1.82)
***Child satisfaction T3***			**Max** ***N*** **= 122**	**Max** ***N*** **= 115**	**Max** ***N*** **= 111**				
Somewhat/very much like standing during class?	188	68.6	–	52.6	79.5	0.001	–	–	3.53 (1.69, 7.35)
Easier/much easier to listen/do work when standing?	187	25.1	–	15.8	31.5	0.031	–	–	2.41 (1.08, 5.35)
Would like more standing lessons?	226	67.7	–	63.5	72.1	0.20	–	–	1.48 (0.81, 2.70)
Somewhat/very much like active breaks after sitting?	188	78.2	–	75.9	80	0.58	–	–	1.26 (0.56, 2.82)
Easier/much easier to listen/do work after active break?	188	54.8	–	50.6	58.1	0.34	–	–	1.38 (0.72, 2.65)
Somewhat/very much like active/standing homework?	229	64.6	69.7	53.2	63.3	0.21	0.50 (0.23, 1.07)	0.76 (0.36, 1.61)	1.52 (0.65, 3.54)
Easier/much easier to do homework when standing/active?	232	40.5	48.7	17.8	41.2	0.008	0.24 (0.10, 0.59)	0.75 (0.38, 1.47)	3.14 (1.18, 8.35)
***Child satisfaction T4***			**Max** ***N*** **= 139**	**Max** ***N*** **= 91**	**Max** ***N*** **= 102**				
Somewhat/very much like standing during class?	142	78.2	–	86.6	70.7	0.038	–	–	0.37 (0.15, 0.95)
Easier/much easier to listen/do work when standing?	141	27	–	16.4	36.5	0.018	–	–	2.93 (1.20, 7.14)
Would like more standing lessons?	189	61.9	–	58.9	64.6	0.63	–	–	1.19 (0.59, 2.40)
Somewhat/very much like active breaks after sitting?	132	83.3	–	84.5	82.4	0.66	–	–	0.78 (0.27, 2.28)
Easier/much easier to listen/do work after active break?	136	46.3	–	37.7	53.3	0.12	–	–	1.80 (0.86, 3.75)
Somewhat/very much like active/standing homework?	107	57	58.3	52.4	57.7	0.92	0.79 (0.22, 2.83)	0.83 (0.25, 2.83)	1.06 (0.25, 4.48)
Easier/much easier to do homework when standing/active?	106	49.1	47.5	47.6	53.8	0.90	1.10 (0.33, 3.66)	1.33 (0.40, 4.47)	1.21 (0.29, 5.08)
^a^Would like more active breaks?	193	71.5	–	67	75.5	0.24	–	–	1.50 (0.76, 2.96)

Total N and N Yes = Intervention groups combined. *PA-I* Physical activity intervention group, *SB-I* Sedentary behaviour intervention group, SB + PA-I=Combined physical activity and sedentary behaviour group. T3: 18-months (Nov-Dec 2011). T4: 30-months (Nov-Dec 2012). ^a^Bootstrapped logistic mixed models adjusted for school SES. Empty cells relate to questions not being asked of that group due to lack of relevance

### Sustainability (Teachers Qualitative Data)

Qualitative data from T3 and T4 teacher surveys and lesson evaluations captured program sustainability and integration of standing lessons and active breaks into school policy. Themes were broadly consistent at T3 and T4 and across intervention groups (Table 4).

#### Facilitators to program sustainability

At T3, teacher awareness of program benefits to the children and teaching practice, was the dominant theme associated with program sustainability:

“*We are all used to it and it doesn’t impact on the classroom time etc but helps to transition to new lessons or refocus on a task! We all need the break*!” [T3, Grade 3/4 Teacher, SB + PA-I]

This was followed by children’s enjoyment, which encouraged teachers to continue implementation. At T4, five teachers inferred that additional ideas and program materials to support delivery would facilitate sustained implementation. At the organisational level, effective program integration into existing teaching practices was the key theme associated with sustainability at the school level. Specifically, teachers referred to the importance of program integration into other school and curriculum areas, including regular professional development and demonstrations of implementation. At T4 in particular, school leadership and management support was perceived as integral to long-term integration of the program as a whole-of-school approach:

“*School leadership should ensure that all students across the school are involved in active learning tasks every day!*” [T4, Grade 6 Teacher, PA-I]

One teacher at T3, and two teachers at T4, referred to prioritising and raising the profile of physical activity within schools, as integral to program sustainability.

#### Facilitators to integration of standing lessons and active breaks into school policy

Integrating and prioritising inclusion of *TransformUs* into school and curriculum planning meetings, including prioritising the program within a supportive planning strategy, was identified as the main facilitators to school policy integration at an organisational level:

“*Needs* [standing lessons] *to be integrated in the curriculum from the beginning of the year and done all the way through the school*.” [T4, Grade 3 Teacher, SB-I]

At the individual level, promoting and raising awareness of the program values and benefits among teachers was the primary theme associated with school policy integration. This included re-framing *TransformUs* outcomes to include health and educational gains for the School Council. Incorporating the program into teacher training and professional development sessions was described as facilitating whole-of-school adoption. Six teachers (one at T3 and five at T4) described school reinforcement of teacher commitment to, and staff support for program delivery, was necessary for policy integration. Specifically this included a cultural and environmental shift in classroom management:

“*Again, all PLT* [Professional Learning Team] *members must attempt to integrate it* [active breaks] *into the culture/everyday environment of the classroom*.” [T4, Teaching Grade unknown, SB + PA-I]

#### Barriers to program sustainability

At an organisational level, time was the most frequently occurring barrier to long-term program implementation, followed by the perception that the nine key messages were insufficiently integrated across the broader school curriculum. One teacher referred to a lack of consistent reinforcement and awareness of the program at the organisational level, which inhibited continued delivery:

“*Great to have more physical activity, however unless it’s discussed and encouraged regularly, it gets put on the back burner. So much happens each day that programs get pushed back. Unfortunately this happened with TransformUs at* [name of school].” [T4, Grade 5 Teacher, SB-I]

Far fewer barriers were reported at the individual teacher level. Perceived demands of complete program delivery and perceived challenges of effective integration into existing practices hindered sustainability amongst nine teachers at T4, and one teacher at T4.

#### Barriers to integration of standing lessons and active breaks into school policy

The most frequently occurring barrier to policy integration, was the perception that a mandated policy relating to *TransformUs* was not required. Reasons included the practical challenges with classroom infrastructure (e.g. size), time constraints with the crowded curriculum, and difficulties gaining whole of school and committee support for a new policy:

“*For teachers it* [active breaks] *would be easy. As for policy, getting things made into “school policy“ is difficult as it goes through council*.” [T4, Grade 5/6 Teacher, PA-I]

### Associations between implementation level and outcomes

At T3, 48% of teachers were significantly more likely to report moderate implementation (delivering approximately two-thirds of the entire intervention) than low (26%, *p* = 0.03) and high (26%, *p* = 0.03) implementation (Additional File 5). At T4, 46% of teachers delivered approximately one-third of the entire intervention (low level of implementation). At T4, teachers were significantly less likely to report a level of implementation classified as high (11%), than both low (46%, *p* = 0.003) and moderate (43%, *p* = 0.007) implementation (Additional File 5). There were no statistically significant associations between intervention implementation level and children’s physical activity and sedentary time on an average day, average weekday or average weekend day (Table 9).

**Table 9:** Linear mixed models of associations between teacher implementation score (continuous outcomes) and child physical activity and sedentary behaviour outcomes (combined intervention groups)

Outcome variable (mins/day)	T3			T4		
***T3***	N	B (95% CI)	***p***-value	N	B (95% CI)	***p***-value
Sedentary time average day	105	−0.75 (−9.51, 8.01)	0.86	86	6.17 (−4.16, 16.50)	0.23
Sedentary time weekday	143	−0.23 (−8.69, 8.23)	0.96	125	8.18 (−0.79, 17.14)	0.07
Sedentary time weekend day	134	−2.27 (−16.77, 12.23)	0.75	100	1.43 (−12.37, 15.24)	0.83
Light-intensity physical activity average day	105	−2.26 (−8.51, 3.98)	0.47	86	−4.95 (−12.98, 3.08)	0.22
Light-intensity physical activity weekday	143	−3.18 (− 10.39, 4.04)	0.38	125	−6.96 (− 14.58, 0.66)	0.07
Light-intensity physical activity weekend day	134	−2.59 (111.02, 5.83)	0.54	100	−4.80 (−13.82, 4.23)	0.29
MVPA average day	105	1.22 (−3.05, 5.49)	0.57	86	−1.11 (−7.32, 5.11)	0.72
MVPA weekday	143	3.39 (−0.76, 7.53)	0.11	125	−0.92 (−5.16, 3.32)	0.66
MVPA weekend day	134	1.26 (−5.86, 8.37)	0.72	100	−0.06 (−5.51, 5.39)	0.98
Sedentary breaks (frequency) average day	105	−3.44 (−9.24, 2.37)	0.24	86	−5.97 (−15.32, 3.38)	0.20
Sedentary breaks (frequency) weekday	143	−4.96 (−10.75, 0.9)	0.09	125	− 5.85 (−13.93, 2.22)	0.15
Sedentary breaks (frequency) weekend day	134	−6.36 (−14.03, 1.31)	0.10	100	−8.61 (−18.36, 1.14)	0.08

MVPA: Moderate- to vigorous-intensity physical activity. T3: 18-months (Nov-Dec 2011). T4: 30-months (Nov-Dec 2012). Analyses adjusted for school SEP, average accelerometry wear time, baseline values of the outcome variables and intervention group. In the models where sedentary breaks were included as the outcome variable, analyses were also adjusted for average sedentary time

## Discussion

This process evaluation provides unique insights into factors which may have influenced the implementation and sustained delivery of *TransformUs*; a school-based intervention to increase children’s physical activity and reduce sedentary behaviour. The results indicate that on the whole, *TransformUs* was appropriate for the school setting, and teachers, parents and children were satisfied with the strategies involved. The evaluation involved approximately half of all teachers and almost all children recruited as part of the RCT. There was, however, substantial variability in implementation of the program among responding teachers, which varied both by intervention group and intervention component. For example, the environmental aspects of *TransformUs* were delivered more consistently and frequently compared to the curriculum-related components.

Dose and fidelity of some of the curriculum-related aspects of the program (i.e., delivery of key messages and active/standing homework) declined over time, yet fidelity to pedagogical aspects of the program (i.e., integration of standing lessons and active breaks in the classroom) increased. Consistently, the availability and use of environmental strategies (physical activity/sports equipment in the classroom and playground line markings) remained high throughout the trial. The substantial variability in teacher dose and fidelity in relation to the *TransformUs* protocol is perhaps not unexpected, given the flexibility and autonomy of teaching styles and curriculum delivery that exists within schools. These differences may also simply reflect the necessary conditions for teachers to deliver *TransformUs*. School-based intervention research suggests that variations in teacher implementation are likely [60], and dose and fidelity may change over time [60, 61]. Without adaptation, interventions are more likely to face resistance by the user and require active engagement for delivery [62].

The majority of responding teachers delivered approximately two-thirds of the entire intervention over the course of the trial. This falls within the range of other school-based interventions reporting teacher delivery of between half [63] and three-quarters [64] of the intervention. Level of implementation was highest amongst teachers in the combined SB + PA-I group. Teachers in this group reported greater fidelity to the pedagogical aspects (standing lesson and active break delivery) and increased dosage of environmental components. The SB + PA-I group included the greatest variety of intervention components for delivery. The increased choice of strategies to implement may have enhanced the schools’ and teachers’ autonomy for delivery [65], facilitating implementation overall. Teacher implementation level was, however, unrelated to the time children spent in physical activity or sedentary behaviour. In general, higher levels of implementation are linked to improved behavioural outcomes [60, 66], even though positive outcomes can still be achieved when an intervention is not necessarily delivered as intended [60, 63]. There are several potential interpretations for this finding. It could suggest that there is no minimum threshold for implementation of *TransformUs* strategies for behavioural outcomes to occur; any changes or promotion of movement in schools is beneficial for child physical activity. Alternatively, as we calculated level of implementation based only on aspects of *TransformUs* implementation that related to teacher level delivery (i.e., dose delivered by teachers), we do not know the extent that factors may have influenced intervention impact. For example, effective implementation also includes factors at an organisational level, such as Principal buy-in and support for implementation, yet we were not able to account for this as part of the implementation score and thus do not know if factors at this level were also associated with intervention impact. Lastly, due to the small sample of responding teachers, we were unable to track individual changes in teachers’ implementation of *TransformUs* over time and how this may relate to child outcomes. It is unknown therefore if the lack of associations between implementation levels and outcomes resulted from the lack of sensitivity of the standardised score. Nonetheless, since variations in teacher dose and fidelity were unrelated to child behavioural outcomes in this study, yet the intervention has demonstrated positive effects on children’s’ MVPA and sedentary time [29, 30], the potential translatability of *TransformUs* into a practice context may be increased.

The *TransformUs* key messages and active/standing homework topics were designed in accordance with the existing curriculum, and adaptation was encouraged to suit planned lessons. However, the notable differences in teacher implementation of these curriculum-related components, compared to the pedagogical and environmental components, raises questions about the feasibility of uniformly integrating curriculum-based strategies within the school setting. Previous intervention research in schools has highlighted that intervention fidelity can be compromised due to challenges that teachers face adapting intervention lessons for contextual relevance [67]. Evaluation of the CATCH-ON study in the United States showed that the classroom curriculum and family-based components had the lowest levels of program institutionalisation [68]. Since adaptability is one element of sustainability, the goal for intervention evaluation may be to ascertain what degree of fidelity is needed to achieve outcomes [61], and how much variation is necessary to align with the delivery context [69]. Consistent with previous interventions targeting school settings [70], and a systematic review of factors required for sustainable implementation in schools [71]; the need for school Principals and senior school leaders to champion and embed initiatives within routine school practices, was integral to *TransformUs* uptake, delivery and sustainability. Innovative ways of building schools’ capacity to support teachers to align and integrate curriculum modifications into their existing teaching practices, may improve the feasibility and delivery of programs such as *TransformUs.*

Overall, *TransformUs* was perceived as being appropriate according to teachers, based on low amounts of preparation required to implement, ease of delivery and integration into teachers’ current learning themes. Qualitative data also highlighted that effective integration of *TransformUs* into existing practices was the main facilitator to consistent delivery and sustained implementation, and would facilitate successful integration of all aspects of the program into school policy. Compatibility of an intervention to the context [32], and effective integration into existing organisational routines, including setting appropriateness, are well-documented precursors to more effective implementation [46, 62, 72]. Understanding the delivery context can help explain the conditions necessary for optimal implementation, and the intervention’s generalisability to other settings and the reasons why variances in fidelity and adaptation may occur [32].

Variations in program implementation over time may also reflect differences in teacher exposure to training and implementation support between baseline and T4. During intervention years one (baseline) and two (corresponding to the T3 data collection), *TransformUs* teachers received a half-day training session and a follow-up mid-year morning tea to discuss and solve challenges to implementation. In the final year of the trial (corresponding to T4 data collection), teachers received training materials at the beginning of the year, but no face-to-face training. In addition, some teachers may have been exposed to training during only one school year, whereas others may have been exposed to training over multiple years. This would have depended on which grades they taught over the three school years the intervention was implemented. Increased participation in training has been associated with improved implementation of a school-based physical activity intervention [73], and a supporting infrastructure that builds capacity in individuals is central to implementation [46, 74].

Consistent with previous literature on factors influencing implementation of school-based physical activity interventions [75], qualitative data suggested that teacher awareness of *TransformUs* values and benefits, and children’s enjoyment were related to implementation. Perceived barriers included a lack of school-level awareness and promotion, organisational level endorsement and whole-of-school support, time constraints and a lack of program integration into existing school practices. Organisational climate and level of institutionalisation have previously been linked to the degree of implementation of school-based interventions [73]. Consistent with this literature, *TransformUs* teachers identified whole-of-school leadership, prioritisation, support and commitment to delivery, as facilitators to school policy integration. If interventions are to have a more substantial impact on children’s behaviours, implementation strategies need to target changes in the organisational infrastructure and culture of schools, in equal measure to changing observable behaviours.

In terms of the dose received among children and parents, consistently across all intervention components, reported receipt and/or awareness of *TransformUs* was lower at T4 than T3, although the dose received among children varied by intervention component. Consistent with previous research exploring dose received of a school-based obesity prevention intervention [76], potentially, some *TransformUs* components may have been easier to adopt by the children than others, explaining the differences in dose received. For example, some *TransformUs* elements were delivered as a whole-of-class activity increasing the likelihood of participation (e.g., taking part in a standing lesson), compared to other program elements that required individual choice to adopt (e.g., using physical activity/sports equipment during recess and lunchtime) or environmental awareness (e.g., seeing the promotional signage). Nonetheless, whilst teachers reported increased implementation of the pedagogical aspects of *TransformUs* over time, children’s recall of these aspects reduced. There is the potential that changes in children’s recall may reflect that the classroom components increasingly became part of routine practice and thus less ‘novel’ than when implemented in earlier phases of the trial. Previous studies of school-based interventions have suggested that students’ characteristics, such as their attitudes, motivation and engagement towards an intervention, can influence dose received [75, 77, 78], due to the extent that they participate. Whilst *TransformUs* was positively received by parents and children, future studies of *TransformUs* could explore any relationships between child and parent satisfaction and reported dose received.

A core aim of this evaluation was to understand teacher-led delivery of *TransformUs* and whether this was associated with outcomes. Teacher ‘implementation level’ was based on teacher adherence to intended delivery of *TransformUs* (fidelity) and the proportion and frequency of components implemented (dose delivered). This is consistent with previous conceptualisations of implementation in process evaluation [47], and studies which have associated implementation level with outcomes [57]. There are, however, many multi-level factors at the individual, organisational and macro level (such as school culture and characteristics) which are associated with effective implementation of interventions in the school setting [35]. Level of implementation can also include the optimal ‘dose received’ by participants [79], in addition to primarily dose delivered that was used in this study. Future research which explores the interaction of multi-level implementation factors on outcomes of school-based interventions, would improve our understanding of the conditions required for effective implementation and the extent that these factors mediate outcomes.

### Strengths and limitations

Strengths of this study include assessment of numerous aspects of implementation at multiple time points to understand when optimal implementation may occur. Implementation processes are not static, rather they change to reflect the context within which implementation occurs. Implementation of some components of *TransformUs* (e.g., key messages and active/standing homework) was greater at T3 than T4, but the reverse was true for other components (e.g., integration of standing lessons and active breaks in the classroom). Had the changes in delivery over time not been captured, the conclusions may have over- or under-estimated the level of implementation delivered and received. The collection of both quantitative and qualitative survey data provided a more in-depth understanding of participants’ experiences. The qualitative data in particular provided insight into reasons for the variations in implementation; and have directly informed strategies to support delivery and sustainability of the program for future roll-out.

This study is not without limitations. Due to small numbers of teachers, models in Table 3 were not adjusted for school clustering or SES, and thus the results should be interpreted with caution. The small sample of teachers also meant exploration of the associations between implementation levels and outcomes was limited by use of a standardised score, and tracking of individual changes was not possible. The standardised implementation score may not have been sufficiently sensitive to detect any differences in implementation between groups. Strategies to maximise participant retention in the evaluation would have enabled more in-depth assessment of the relationship between implementation quality and program impact. Assessment of teacher implementation was based on teacher self-report of the previous school term, which is subject to recall bias. As only a sample of all participating teachers provided this self-report data, we do not know the level of implementation from non-responding teachers. Direct observations of implementation dose and fidelity may have improved accuracy of implementation assessment [67]. Lastly, our assessment of parent dose received included the *number* of program newsletters parents reported receiving. We were unable to assess whether parents read or acted on recommendations in these newsletters. This is an additional, important layer of assessing intervention and implementation effectiveness of programs such as *TransformUs* and others. Future evaluations of interventions that wish to ascertain dose received, would benefit from adopting multiple different indicators for this construct.

## Conclusions

This study demonstrated that intervention dose and fidelity increased over time, and that children’s enjoyment, senior school leadership and effective integration of interventions into school practices facilitated improved intervention delivery and sustainability. Teacher level of implementation and child behavioural outcomes were unrelated, suggesting intervention efficacy was achieved irrespective of implementation variability. The potential translatability of *TransformUs* into practice contexts may therefore be increased. Findings have informed the scale up of *TransformUs* across Victoria, Australia.

## Supplementary Information

**Additional file 1. **Intervention components and corresponding intervention arms. Theoretical basis of *TransformUs* intervention components and objectives. Alignment of *TranformUs* intervention components to the three intervention arms, and how the social cognitive theory, behavioral choice theory and ecological systems theory correspond to the intervention components.

**Additional file 2. ***TransformUs* TIDieR Checklist.

**Additional file 3. ***TransformUs* teacher survey SB + PA-I at T3. Copy of the TransformUs teacher survey for the SB + PA-I group at data collection time point 3.

**Additional file 4.** Participant response rates at T3 and T4. Teacher, parent and child response rates to evaluation surveys at T3 and T4 and teacher response rates to lesson evaluations during 2010 and 2011.

**Additional file 5. **Distribution of teachers and children by level of implementation by intervention group. Distribution of teachers and children based on the percentage of the intervention delivered (low < 33%, moderate > 33 < 67% or high level > 67%of implementation).

## Data Availability

The datasets used and/or analysed during the current study are available from the corresponding author on reasonable request.

## Abbreviations

C-: Control group
CI: Confidence interval
METs: Metabolic equivalents
MVPA: Moderate- to vigorous-intensity physical activity
PA: Physical activity
PA-I: Physical activity intervention group; SB Sedentary behaviour
SB-I: Sedentary behaviour intervention group
SB + PA-I: Sedentary behaviour and physical activity intervention group
SEIFA: Australian bureau of statistics socio-economic indexes for area
SEP: Socioeconomic position
