# Supplementary material for: Is level of implementation linked with intervention outcomes? Process evaluation of the TransformUs intervention to increase children’s physical activity and reduce sedentary behaviour
Source: Int J Behav Nutr Phys Act. 2022 Sep 17;19:122. doi: 10.1186/s12966-022-01354-5 (PMC9482275; doi:10.1186/s12966-022-01354-5)
Supplement: Supplementary file 1 — Additional file 1. Intervention components and corresponding intervention arms. Theoretical basis of TransformUs intervention components and objectives. Alignment of TranformUs intervention components to the three intervention arms, and how the social cognitive theory, behavioral choice theory and ecological systems theory correspond to the intervention components. [file 12966_2022_1354_MOESM1_ESM.docx]

**Additional File 1**

***TransformUs* intervention components by intervention arm**

|  | **Intervention component** | **Intervention group** | | |
| --- | --- | --- | --- | --- |
|  |  | PA-I | SB-I | SB+PA-I |
| **School setting** | Curriculum component | 18 key PA messages incorporated into classroom lessons (9 per year) | 18 key SB messages incorporated into classroom lessons (9 per year) | 18 key PA & SB messages incorporated into classroom lessons (9 per year) |
|  | Lesson delivery | NA | Standing lessons (1 × 30-min/day)  Active 2- min breaks after 30-min class time | Standing lessons (1 × 30-min/day)  Active 2- min breaks after 30-min class time |
|  | Physical environment | Provision of PA/sports equipment, line markings and signage  Provision of pedometers | Standing easels (1 x 30min/day) | Standing easels (1 x 30min/day)  Provision of PA/sports equipment, line  markings and signage  Provision of pedometers |
| **Family setting** | Homework tasks | Tasks to incorporate PA while completing homework | Tasks to reduce SB while completing homework | Tasks to incorporate PA and reduce SB while completing homework |
|  | Newsletters | Tips to promote PA at home  Opportunities to be active in the home and neighbourhood | Tips to reduce sitting time at home | Tips to reduce sitting time and promote at PA home |

PA = Physical activity, SB = Sedentary behaviour, PA-I = Physical activity intervention group, SB-I = Sedentary behaviour intervention group, SB+PA-I = Combined physical activity and sedentary behaviour group

**Theoretical basis of *TransformUs* intervention components and objectives**

| **Constructs** | **Mediators or determinants** | **Program Objectives** |
| --- | --- | --- |
| **Intrapersonal**  Confidence  Preference  Expectations  Expectancies  Skills  Behavioral rehearsal | Self-efficacy  Enjoyment  Benefits/barriers  Evaluation of anticipated outcome  Self-management  Self-monitoring & contracting | Improve confidence in ability to be active or reduce sedentary time  Increase enjoyment and preference for physical activity  Increase knowledge of benefits & strategies to overcome barriers  Alter perception of pros and cons of being more active  Self-rewards, self-instructions, TV viewing styles  Goal setting, contracting with others, rewards |
| **Interpersonal**  Observational learning  Social support  Social structure | Modelling by parents/siblings  Modelling/social support  Rules | Encourage parents & siblings to reduce their own SB & increase PA  Encourage parents & siblings to support child to spend less time in SB & more time in PA; teachers encourage/support PA during recess/lunch  Parents enforce rules regarding limiting screen time at home, during meals, during daylight hours |
| **Environmental**  Imposed environment  Imposed environment  Imposed environment | Availability  Access  Policy | Increase the amount of PA equipment available at school & home. Reduce the availability of TVs/computers/electronic games at home  Increase access/opportunities for PA at school and at home. Decrease access to TV/computers/electronic games at home  Interrupted sitting during class-time; presence of supervising teachers during recess/lunch |

Table sourced from Salmon et al. 2011 ‘A cluster-randomized controlled trial to reduce sedentary behavior and promote physical activity and health of 8-9 year olds: The Transform-Us! Study’. *BMC Public Health*. Theories include social cognitive theory (26), behavioural choice theory (27) and ecological systems theory (28). PA= Physical activity; SB = Sedentary Behaviour.
