# Supplementary material for: Is level of implementation linked with intervention outcomes? Process evaluation of the TransformUs intervention to increase children’s physical activity and reduce sedentary behaviour
Source: Int J Behav Nutr Phys Act. 2022 Sep 17;19:122. doi: 10.1186/s12966-022-01354-5 (PMC9482275; doi:10.1186/s12966-022-01354-5)
Supplement: Supplementary file 3 — Additional file 3. TransformUs teacher survey SB + PA-I at T3. Copy of the TransformUs teacher survey for the SB + PA-I group at data collection time point 3. [file 12966_2022_1354_MOESM3_ESM.pdf]

FOR OFFICE USE ONLY

ID:

DATE RECEIVED:

GROUP: 4

TRANSFORM-US! TEACHER: 2010/2011

DEAKIN UNIVERSITY

TRANSFORM - US!

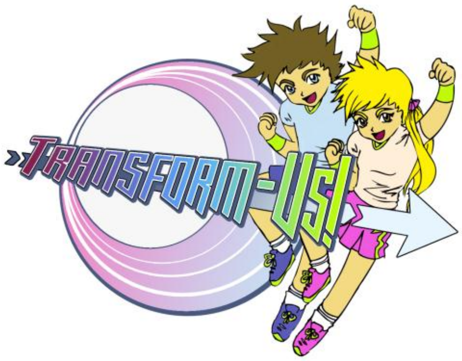

TEACHER QUESTIONNAIRE
Term 4, 2011

Your name: ______________________________

Your class: ______________________________

School: ______________________________

Today's date: __ __ / __ __ / 20__ __

If you have any questions, please contact 9244 6278

IMPORTANT INSTRUCTIONS – PLEASE READ

Thank you for taking the time to complete this survey. It will take you approximately 15 minutes to complete, although this might vary depending on your answers. Once you have finished your survey, please place it in the envelope provided and we will collect it from your school.

Please answer each question by ticking or circling the most suitable option. Where you are asked to write an answer please read the question carefully and answer the best you can in the space provided. If you are unsure about how to answer a question, please choose the answer that best reflects how you feel.

When marking your answers on the survey, please clearly tick or circle your response so we can easily see which answer you chose. For example:

When asked to tick your answer, please do so like this:

Strongly disagree$_1$	Disagree$_2$	Neither agree or disagree$_3$	Agree$_4$	Strongly agree$_5$
○	○	○	○ ✓	○

If you make an error, please clearly cross out the incorrect answer and tick the correct answer. For example:

Strongly disagree$_1$	Disagree$_2$	Neither agree or disagree$_3$	Agree$_4$	Strongly agree$_5$
○	○ ✓	○	○ ✓ ✗	○

Important definitions:

Physical Education (PE): Often termed 'Health and Physical Education', PE is the area of the curriculum that provides students with knowledge, skills and behaviours to enable them to achieve a degree of autonomy in developing and maintaining their physical, mental, social and emotional health. This domain focuses on the importance of a healthy lifestyle and physical activity in the lives of individuals and groups in our society.

Physical activity (PA): is any bodily movement produced by skeletal muscle contraction that increases energy expenditure. For example, walking, running, playing soccer and dancing.

ID:

YOUR STUDENTS, CLASSROOM and SCHOOL

1. In a typical week, during the CURRENT SCHOOL TERM, which of the following physical activities do your students usually participate in during class time and for how long?

Activity	Do your students usually do this activity? No$_1$	Yes$_2$	How many times in a week?	How many TOTAL minutes per week?
EXAMPLE Sport	○	✓	2	60
a. PE (Health and Physical Education)	○	○	___________	___________
b. Sport (organised games)	○	○	___________	___________
c. Fitness activities eg Huff and Puff	○	○	___________	___________
d. Active play during class time	○	○	___________	___________
e. Other ____________________	○	○	___________	___________

2. How often do you deliver lessons that require children to stand up and move around during class? (e.g. plan activity stations around the classroom that the children are required to move between) (please tick *one* response)

Never$_1$	Once/week$_2$	Several times/ week$_3$	Once/day$_4$	Several times/ day$_5$
○	○	○	○	○

3. **In a typical week during the CURRENT SCHOOL TERM, during class lessons, what equipment can you access and what do you use with your children?** (Please tick all that are applicable)

		a. **I can access**		b. **I use**	
		**No$_1$**	**Yes$_2$**	**No$_1$**	**Yes$_2$**
a.	Balls	○	○	○	○
b.	Bats	○	○	○	○
c.	Frisbees	○	○	○	○
d.	Bean bags	○	○	○	○
e.	Music	○	○	○	○
f.	Standing Easels	○	○	○	○
g.	Clipboards	○	○	○	○
h.	Playground markings (eg. 4-square)	○	○	○	○
i.	Sporting equipment	○	○	○	○
j.	Other _______________	○	○	○	○

4. **How much do you agree with the following statements?** (please tick *one* response per line)

		**Strongly disagree$_1$**	**Disagree$_2$**	**Neither agree or disagree$_3$**	**Agree$_4$**	**Strongly agree$_5$**
a.	School plays an important role in children's overall physical activity by providing opportunities to be active	○	○	○	○	○
b.	School plays an important role in children's overall physical activity through teaching and learning	○	○	○	○	○
c.	The classroom teacher plays an important role in children's overall physical activity, by providing opportunities for them to be active	○	○	○	○	○
d.	The classroom teacher plays an important role in children's overall physical activity, through teaching and learning	○	○	○	○	○
e.	Children should do at least one hour of physical activity every day	○	○	○	○	○
f.	Children need to sit during lessons to learn effectively	○	○	○	○	○
g.	Boys in my class spend most of their recess/lunch breaks running around and being active	○	○	○	○	○
h.	Girls in my class spend most of their recess/lunch breaks running around and being active	○	○	○	○	○

i. Children should spend less than 2 hours per day watching TV, using computers or playing electronic games	○	○	○	○	○
j. Children would be too disruptive when standing up during lessons	○	○	○	○	○
k. It would be difficult to settle children down if they move around or stand up during class	○	○	○	○	○
l. If children's homework incorporated a physical activity component, I would see it as important for their educational development (eg 'measurement' homework using their steps/pace as the unit of measuring)	○	○	○	○	○
m. It is important that parents reinforce the key learnings that teachers deliver to students	○	○	○	○	○
n. The classroom teacher plays an important role in ensuring children are sufficiently active at school	○	○	○	○	○

5. How much do you agree with the following statements? (please tick *one* response per line)

	**Strongly disagree** 1	**Disagree** 2	**Neither agree or disagree** 3	**Agree** 4	**Strongly agree** 5
a. I don't have time to include physical activity into my lesson/class time	○	○	○	○	○
b. I don't know how to keep the children active when I take them outside	○	○	○	○	○
c. The fear of litigation puts me off doing physical activities with my class	○	○	○	○	○
d. The curriculum is too crowded to include physical activity	○	○	○	○	○
e. Being active is not an important part of the curriculum	○	○	○	○	○
f. I am not confident teaching <u>physical activity</u> to children	○	○	○	○	○
g. I am not confident teaching <u>physical education</u> to children	○	○	○	○	○
h. The literacy and numeracy block restricts time for physical activity	○	○	○	○	○
i. Time spent in physical activity might reduce academic performance levels in the school	○	○	○	○	○
j. Time spent in physical activity might reduce academic performance of some children	○	○	○	○	○
k. I am too old/unfit to teach physical activities	○	○	○	○	○

l. I am not interested in incorporating physical activity into classes	○	○	○	○	○
m. Getting children moving during class time will be too disruptive to the class	○	○	○	○	○
n. Getting children moving during class time will be too disruptive to their learning	○	○	○	○	○
o. I prefer children to sit quietly during class time	○	○	○	○	○

6. Please tell us how often your students' receive homework during the CURRENT SCHOOL TERM. (Please tick *one* response per line)

Never$_1$	Once/ Month$_2$	Once/ Fortnight$_3$	1-2 days /week$_4$	3-4 days /week$_5$	Everyday$_6$	Don't know$_7$
○	○	○	○	○	○	○

7. Please tell us how often your students' homework requires him/her to be active during the CURRENT SCHOOL TERM. (Please tick *one* response per line)

	Never$_1$	Once/ Month$_2$	Once/ Fortnight$_3$	1-2 days /week$_4$	3-4 days /week$_5$	Everyday$_6$	Don't know$_7$
a. My students' homework requires him/her to be active	○	○	○	○	○	○	○

8. How supportive are the following for providing opportunities for your class to be physically active? (please tick *one* response per line)

	Very supportive$_1$	Somewhat supportive$_2$	Not at all supportive$_3$	Don't know$_4$
a. Principal	○	○	○	○
b. PE teacher	○	○	○	○
c. School Council	○	○	○	○
d. Curriculum Committee	○	○	○	○
e. Parents	○	○	○	○
f. Other: ________________________	○	○	○	○

9. Is sporting equipment available for students to use during recess and lunchtime?

☐1 Yes

☐2 No

10. Is sporting equipment available for students to use at other times?

☐1 Yes

☐2 No

11. During recess and lunch breaks, while on duty, I…

	Never 1	Once/ fortnight 2	1-2 times/ week 3	3-4 times/ week 4	Every day 5
a. Make sports equipment available to all children	○	○	○	○	○
b. Encourage children to be physically active	○	○	○	○	○
c. Help children to organise active games	○	○	○	○	○
d. Other (please specify) ______________________	○	○	○	○	○

12. During recess and lunch breaks, other teachers on duty…:

	Never 1	Once/ fortnight 2	1-2 times/ week 3	3-4 times/ week 4	Every day 5
e. Make sports equipment available to all children	○	○	○	○	○
f. Encourage children to be physically active	○	○	○	○	○
g. Help children to organise active games	○	○	○	○	○
h. Other (please specify) ______________________	○	○	○	○	○

THE LEARNING ENVIRONMENT

The next question asks about interrupted classroom lessons. These are standard curriculum lessons delivered to children in a way that reduces the time they spend sitting. For example, the children stand and act out story characters while completing their reading comprehension lesson.

13. How much do you agree or disagree with the following statements? (Please tick *one* response per line)

	Strongly agree₁	Agree₂	Neither₃	Disagree ₄	Strongly disagree₅
a. Interrupted classroom lessons would be too disruptive to the class	○	○	○	○	○
b. Interrupted classroom lessons would increase children's ability to complete the task	○	○	○	○	○
c. Interrupted classroom lessons would negatively affect academic outcomes	○	○	○	○	○
d. Interrupted classroom lessons would result in children losing concentration	○	○	○	○	○
e. Children would be too disruptive to the learning environment when standing up during class	○	○	○	○	○

14. How confident are you that that you could….. (Please tick *one* response per line)

	Not at all sure₁	A bit sure₂	Fairly sure₃	Quite sure₄	Very sure₅
a. Modify class lessons to increase children's physical activity during class time	○	○	○	○	○
b. Assist children to be active during recess and lunch breaks	○	○	○	○	○
c. Engage parents to reinforce physical activity messages at home	○	○	○	○	○
d. Modify children's homework to incorporate more physical activity	○	○	○	○	○
e. Engage parents in homework tasks that had an active component	○	○	○	○	○
f. Modify class lessons to limit children's sitting time during class	○	○	○	○	○
g. Modify children's homework to require less sitting	○	○	○	○	○

ABOUT YOU

15. How old are you? _________ years

16. What is your sex? (please tick *one* response)

☐$_1$ Male

☐$_2$ Female

17. Are you currently: (please tick *one* response)

☐$_1$ Employed full time

☐$_2$ Employed part time

☐$_3$ Other (please specify) ________________________

18. How long have you been teaching primary school children? _________ years

19. Did you attend the Transform-Us! Professional Development (PD) session in 2010?

☐$_1$ Yes

☐$_2$ No

20. Did you attend the Transform-Us! PD session in 2011?

☐$_1$ Yes

☐$_2$ No

21. Did you attend the Transform-Us! morning tea catch-up in 2011?

☐$_1$ Yes

☐$_2$ No

The following questions exclude the Transform-us! PD session

22. <u>Since you last completed a Transform-Us! survey</u>, have you undertaken formal education or training (eg Professional Development (PD)/ University Degree) specifically for Physical Education delivery among young children? (Please tick *one* response)

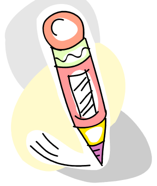

☐$_1$ Yes

☐$_2$ No *(if 'No' please go to Question 24)*

23. How long did the training go for? (Please tick *one* response)

☐1 Less than 1 day (e.g. half a day or a few hours)

☐2 1-2 days

☐3 3 days - 3 months

☐4 3 months - 12 months

☐4 12 months or longer

24. <u>Since you last completed a Transform-Us! survey</u>, have you undertaken formal education or training (eg PD) which focuses on physical activity for young children? (Please tick *one* response)

☐1 Yes

☐2 No *(if 'No' please go to question 26)*

25. How long did the training go for? (Please tick *one* response)

☐1 Less than 1 day (e.g. half a day or a few hours)

☐2 1-2 days

☐3 3 or more days

26. How much do you agree with the following statements? (please tick one response per line)

	Strongly disagree 1	Disagree 2	Neither agree or disagree 3	Agree 4	Strongly agree 5
a. I enjoy being physically active	○	○	○	○	○
b. It is important to me that I do not spend too much time watching TV/videos/DVDs/ using the computer during leisure time	○	○	○	○	○
c. It is important to me that I do enough physical activity each week	○	○	○	○	○

27. In the current school term, do you usually participate in at least 30 minutes of moderate to vigorous intensity physical activity 5 or more days of the week? (eg. brisk walk, jogging, organised sport)

(Please tick *one* response)

☐1 Yes

☐2 No

ABOUT Transform-Us!

28. Have you heard of the Transform-Us! program?

☐1 Yes

☐2 No

a. If yes, what do you know about it?

____

____

____

____

29. Have your students parents or other parents at the school spoken to you about the Transform-Us! program?

☐1 Yes

☐2 No

a. If yes, what sorts of things did they talk about?

____

____

____

____

Key Messages/Lessons

In your professional development session we provided you with nine key messages/lessons to be delivered to the children. The following questions are about these key messages/lessons.

30. Did you deliver the following key messages/lessons to the students in 2011? *(please tick one response per line)*

		Yes1	No2
a.	Health impacts of sedentary behaviour (sitting time) and physical activity	○	○
b.	Self awareness and management of their sitting time and physical activity (when they do it)	○	○
c.	The school and home environments in which they are physically active and where they sit	○	○
d.	Creative thinking – creating new active games as alternatives to sitting	○	○
e.	Decision making - making active/less sedentary decisions when faced with active and less active scenarios/case studies	○	○
f.	Step challenge to calculate the number of steps to locations in the school area	○	○

g.	Overcoming barriers and increasing confidence to be active when faced with barriers eg it's raining	○	○
h.	Active parent and teacher role models	○	○
i.	Families being active together	○	○

31. If you did not deliver all key messages/lessons, please let us know why?

____

____

____

____

32. Please rate your response to each of the statements below *(please tick one response per line)*

Please rate:	**1**	**2**	**3**	**4**	**5**	**6**	**7**	**8**	**9**	**10**
a. The amount of preparation required to deliver the key messages/lessons (*1 = very little, 10 = a lot)*	○	○	○	○	○	○	○	○	○	○
b. How easy the key messages/lessons were to deliver *(1 = very easy, 10 = very difficult)*	○	○	○	○	○	○	○	○	○	○
c. How well the key messages/lessons were integrated into the current learning theme of your class *(1 = poorly integrated, 10 = very well integrated)*	○	○	○	○	○	○	○	○	○	○
d. The children's level of engagement during the key messages/lessons *(1= not engaged at all,10= much more strongly engaged)*	○	○	○	○	○	○	○	○	○	○
e. The children's enjoyment of the key messages/lessons *(1= did not enjoy it at all, 10= greatly enjoyed it)*	○	○	○	○	○	○	○	○	○	○

Equipment and signage

In your professional development session we provided you with sporting equipment and signage to encourage children to be active during recess and lunch breaks. Line markings on the school grounds were also provided. The following questions are about this equipment, signage and line markings.

33. Was the sporting equipment provided made freely available to all students at recess and lunch times?

☐1 Yes

☐2 No

a. If no, what rostering system was used? (e.g. equipment allocated to different a level on different days etc)

____

____

____

____

34. Did you use the sporting equipment provided during class time?

☐1 Yes

☐2 No, *please continue to q36*

35. How often did you use the sporting equipment during class time?

Never 1	Once/ fortnight 2	1-2 times/ week 3	3-4 times/ week 4	Every day 5
○	○	○	○	○

36. Was the signage provided by Transform-Us! visible to students at recess and lunch times?

☐1 Yes

☐2 No

a. If no, why not?

____

____

____

____

37. Did you use the ground line markings (e.g. 4-square courts, coloured markings) provided by Transform-Us! during class time?

☐1 Yes

☐2 No, *please continue to q39*

38. How often did you use the line markings during class time? *(please tick one response)*

Never 1	Once/ fortnight 2	1-2 times/ week 3	3-4 times/ week 4	Every day 5
○	○	○	○	○

39. Do you have any suggestions of equipment/signage provisions that would assist children to be active during recess and lunch times?

____

____

____

____

40. Did you receive any Sports for Schools equipment (Coles vouchers)?

☐1 Yes

☐2 No, *please continue to q43*

41. Was the Sports for Schools equipment available to the grade 4 students at recess/lunchtime

☐1 Yes

☐2 No

42. Was the Sports for Schools equipment available to the grade 4 students during PE lessons?

☐1 Yes

☐2 No

Standing lessons

In your professional development session we provided suggestions about 'Standing lessons' where you deliver a 30-minute class to the children who are standing. The following questions are about these standing lessons.

43. Were you successful in getting children to complete one standing lesson per day?

☐1 Yes

☐2 No

a. If yes, how did you complete the standings lessons? For example, did you complete a standing lesson in a 30 minutes block, in 3x10 minute blocks etc?

____

____

____

____

b. Why do you think you were successful or unsuccessful in doing this?

____

____

____

____

44. Please rate your response to each of the statements below *(please tick one response per line)*

Please rate:	**1**	**2**	**3**	**4**	**5**	**6**	**7**	**8**	**9**	**10**
a. The amount of preparation required to deliver the standing lessons (*1 = very little, 10 = a lot)*	○	○	○	○	○	○	○	○	○	○
b. How easy the standing lessons were to deliver *(1 = very easy, 10 = very difficult)*	○	○	○	○	○	○	○	○	○	○
c. How well the standing lessons were integrated into the current learning themes of your class *(1 = poorly integrated, 10 = very well integrated)*	○	○	○	○	○	○	○	○	○	○
d. The children's level of engagement during the standing lessons *(1= not engaged at all, 10= much more strongly engaged)*	○	○	○	○	○	○	○	○	○	○
e. The children's enjoyment of the standing lessons *(1= did not enjoy it at all, 10= greatly enjoyed it)*	○	○	○	○	○	○	○	○	○	○
f. The children's concentration after a standing lesson *(1= poor concentration, 10 =high concentration)*	○	○	○	○	○	○	○	○	○	○
g. The children's time-on-task after a standing lesson*(1 =poor time-on-task, 10 =high time-on-task)*	○	○	○	○	○	○	○	○	○	○

45. Would you like to make any additional comments about how easy or difficult the standing lesson strategies were to adapt, prepare and deliver or about how the children received the standing lessons?

____

____

____

____

46. Have you received any comments from your students regarding the standing lessons?

- ☐1 Yes
- ☐2 No, please continue to q48

47. If yes, what sort of comments did you receive - have they been:

- ☐1 generally positive
- ☐2 generally negative
- ☐3 neither positive nor negative

a. Examples of comments:

____

____

____

____

48. Have you received any comments from parents/guardians regarding the standing lessons?

- ☐1 Yes
- ☐2 No if no continue to q50

49. If yes, have comments been:

- ☐1 generally positive
- ☐2 generally negative
- ☐3 neither positive nor negative

a. Examples of comments:

____

____

____

____

50. Do you have any suggestions for improvements to the current list or new standing lesson strategies that could be included among our options from which teachers select?

____

____

____

____

51. Do you think it would be feasible to integrate the standing lesson strategies in classrooms as part of standard school policies?

☐1 Yes

☐2 No

a. If yes, how do you envisage this working?

____

____

____

____

b. If no, please explain why this would not be feasible

____

____

____

____

Active Breaks

In your professional development session we provided you with suggestions about 'active breaks' where children were to perform a 2-minute standing activity to break up prolonged periods of sitting (each 30 minutes). The following questions are about these active breaks.

52. Were you successful in getting children to complete active breaks every 30 minutes (approximately) of sitting?

☐1 Yes

☐2 No, *please continue to q52c*

a. If yes, how long did they go for? ____________________________minutes

b. If yes, what sorts of things did you usually do during the active break?

____

____

____

____

c. Why do you think you were successful or unsuccessful in doing this?

____

____

____

____

53. Please rate your response to each of the statements below *(please tick one response per line)*

Please rate:	**1**	**2**	**3**	**4**	**5**	**6**	**7**	**8**	**9**	**10**
a. The amount of preparation required to deliver lessons with active breaks *(1 = very little, 10 = a lot)*	○	○	○	○	○	○	○	○	○	○
b. How easy lessons with active breaks were to deliver *(1 = very easy, 10 = very difficult)*	○	○	○	○	○	○	○	○	○	○
c. How well the active breaks strategies were integrated into the current learning theme of your class *(1 = poorly integrated, 10 = very well integrated)*	○	○	○	○	○	○	○	○	○	○
d. The children's level of engagement in the lesson, compared to normal lessons without breaks *(1= not engaged at all,10= much more strongly engaged)*	○	○	○	○	○	○	○	○	○	○
e. The children's enjoyment of active breaks *(1= did not enjoy it at all, 10= greatly enjoyed it)*	○	○	○	○	○	○	○	○	○	○
f. The children's concentration after an active break *(1= poor concentration, 10 =high concentration)*	○	○	○	○	○	○	○	○	○	○
g. The children's time-on-task after an active break *(1 =poor time-on-task, 10 =high time-on-task)*	○	○	○	○	○	○	○	○	○	○

54. Would you like to make any comments about how easy/difficult the active break strategies were to adapt, prepare and deliver or about how the children received the active breaks?

____
____
____
____

55. Do you have any <u>suggestions for improvements</u> to the current list of active break strategies that we have suggested?

____
____
____
____

56. Could you <u>suggest any new active break strategies or exercises</u> that could be included among our options from which teachers select?

____
____
____
____

57. Have you received any comments from your students regarding the active breaks?

☐$_1$ Yes
☐$_2$ No, please continue to q59

58. If yes, have comments been:

☐1 generally positive
☐2 generally negative
☐3 neither positive nor negative

a. Examples of comments:

____
____
____
____

59. Have you received any comments from parents/guardians regarding the active breaks?

☐1 Yes
☐2 No, please continue to q61

60. If yes, have comments been:

☐1 generally positive
☐2 generally negative
☐3 neither positive nor negative

a. Examples of comments:

____
____
____
____

61. Did you have any challenges settling students down after completing a standing lesson or active break?

☐1 Yes
☐2 No, please continue to q62

a. How did you manage this?

____
____
____
____

62. Do you think it would be feasible to integrate the active break strategies in classrooms as part of standard school policies?

☐1 Yes
☐2 No

a. If yes, how do you envisage this working?

____
____
____
____

b. If no, please explain why this would not be feasible

____
____
____
____

Homework tasks

In your professional development session we provided suggestions about modifying students' homework so that children complete it while being active/standing. The following questions are about the standing homework.

63. Did you set your students homework to complete while being active/standing?

☐1 Yes

☐2 No

a. If no, why not:

____
____
____
____

64. Have you received any comments from your students regarding the active/standing homework tasks?

☐1 Yes

☐2 No, please continue to q66

65. If yes, have comments been:

☐1 generally positive
☐2 generally negative
☐3 neither positive nor negative

a. Examples of comments:

____
____
____
____

66. Have you received any comments from parents/guardians regarding the active/standing homework tasks?

☐1 Yes

☐2 No, please continue to q68

67. If yes, have comments been:

☐1 generally positive
☐2 generally negative

☐3 neither positive nor negative

a. Examples of comments:

____
____
____
____

68. Have you received any comments from other teachers regarding the active/standing homework tasks?

☐1 Yes

☐2 No

69. If so, have comments been:

☐1 generally positive
☐2 generally negative
☐3 neither positive nor negative

a. Examples of comments:

____
____
____
____

70. Please rate your response to each of the statements below *(please tick one response per statement)*

Please rate:	**1**	**2**	**3**	**4**	**5**	**6**	**7**	**8**	**9**	**10**
a. The amount of preparation required to deliver the active/standing homework tasks *(1 = very little, 10 = a lot)*	○	○	○	○	○	○	○	○	○	○
b. How well the active/standing homework tasks were integrated into the current learning theme of your class *(1 = poorly integrated, 10 = very well integrated)*	○	○	○	○	○	○	○	○	○	○
c. The children's level of engagement in the active/standing homework tasks, compared to normal homework tasks *(1= not engaged at all,10= much more strongly engaged)*	○	○	○	○	○	○	○	○	○	○
d. The children's enjoyment of active/standing homework tasks *(1= did not enjoy it at all, 10= greatly enjoyed it)*	○	○	○	○	○	○	○	○	○	○

71. Please rate the usefulness of the Transform-Us! components listed below *(please tick one response per statement)*

	**Very useful**	**Somewhat useful**	**Not at all useful**
a. Sporting equipment	○	○	○
b. Circus equipment	○	○	○

c. Playground markings	○	○	○
d. Clipboards	○	○	○
e. Standing Easels	○	○	○
f. Morning teas	○	○	○
g. Feedback comparing children's activity levels at the start and end of 2011	○	○	○

72. Will you continue with the Transform-Us! strategies?

☐1 Yes

☐2 No

a. Why/why not

73. Would you recommend Transform-Us! to other teachers?

☐1 Yes

☐2 No

a. Why/why not

74. Are there ways in which Transform-Us! could be improved?

☐1 Yes, *please explain below*

☐2 No

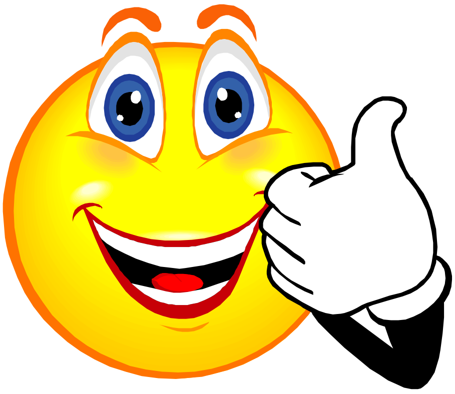

Thank you for completing this survey for us!
