# Supplementary material for: Is level of implementation linked with intervention outcomes? Process evaluation of the TransformUs intervention to increase children’s physical activity and reduce sedentary behaviour
Source: Int J Behav Nutr Phys Act. 2022 Sep 17;19:122. doi: 10.1186/s12966-022-01354-5 (PMC9482275; doi:10.1186/s12966-022-01354-5)
Supplement: Supplementary file 4 — Additional file 4. Participant response rates at T3 and T4. Teacher, parent and child response rates to evaluation surveys at T3 and T4 and teacher response rates to lesson evaluations during 2010 and 2011. [file 12966_2022_1354_MOESM4_ESM.docx]

**Additional File 4. Participant response rates at T3 and T4**

|  | ***T3***  N (%) | ***T4***  N (%) |
| --- | --- | --- |
| Teachers | 60 (56) | 92 (56) |
| Parents | 295 (67) | 246 (82) |
| Children | 418 (98) | 333 (96) |
|  | ***2010 lesson evaluations*** | ***2011 lesson evaluations*** |
| Teachers | 47 (68) | 36 (52) |

Participant response rates to evaluation surveys and lesson evaluations
