# Supplementary material for: Is level of implementation linked with intervention outcomes? Process evaluation of the TransformUs intervention to increase children’s physical activity and reduce sedentary behaviour
Source: Int J Behav Nutr Phys Act. 2022 Sep 17;19:122. doi: 10.1186/s12966-022-01354-5 (PMC9482275; doi:10.1186/s12966-022-01354-5)
Supplement: Supplementary file 5 — Additional file 5. Distribution of teachers and children by level of implementation by intervention group. Distribution of teachers and children based on the percentage of the intervention delivered (low < 33%, moderate > 33 < 67% or high level > 67%of implementation). [file 12966_2022_1354_MOESM5_ESM.docx]

**Additional File 5. Distribution of teachers and children by level of implementation by intervention group**

| **Level of implementation (% entire intervention delivered)** | Intervention group | | | | | | | | | | | | **T3 (%)**  **Teacher Total N=46** | **T4 (%)**  **Teacher Total N=28** |
| --- | --- | --- | --- | --- | --- | --- | --- | --- | --- | --- | --- | --- | --- | --- |
|  | **PA-I** | | | | **SB-I** | | | | **SB+PA-I** | | | |  |  |
|  | **T3 (%)** | | **T4 (%)** | | **T3 (%)** | | **T4 (%)** | | **T3 (%)** | | **T4 (%)** | |  |  |
|  | **Teacher Total N=17** | **Children Total N=123** | **Teacher Total N=13** | **Children Total N=86** | **Teacher Total N=11** | **Children Total N=85** | **Teacher Total N=4** | **Children Total N=18** | **Teacher Total N=18** | **Children Total N=118** | **Teacher Total N=11** | **Children Total N=76** |  |  |
| Low (<33) | 17.7 | 8.9 | 53.6 | 56.9 | 81.8 | 69.4 | 75 | 72.2 | - | - | 27.3 | 18.4 | 26.1 | 46.4 |
| Mod (>33 <67) | 52.9 | 41.5 | 38.5 | 37.2 | 9.1 | 15.3 | 25 | 27.8 | 66.7 | 64.4 | 54.6 | 56.6 | 47.8 | 42.9 |
| High (>67) | 29.4 | 49.6 | 7.7 | 5.8 | 9.1 | 15.3 | - | - | 33.3 | 35.6 | 18.2 | 25 | 26.1 | 10.7 |

PA-I=Physical activity intervention group, SB-I=Sedentary behaviour intervention group, SB+PA-I=Combined physical activity and sedentary behaviour group. PA-I max implementation score=6, SB-I max implementation score=4, SB+PA-I max implementation score=8. T3: 18-months (Nov-Dec 2011). T4: 30-months (Nov-Dec 2012)
